# Supplementary material for: Temporal patterns, spatial risks, and characteristics of tegumentary leishmaniasis in Brazil in the first twenty years of the 21st Century
Source: PLoS Negl Trop Dis. 2023 Jun 7;17(6):e0011405. doi: 10.1371/journal.pntd.0011405 (PMC10281579; doi:10.1371/journal.pntd.0011405)
Supplement: S1 Table — (DOCX) [file pntd.0011405.s001.docx]

**S1 Table. Descriptive measures of new cases of, and deaths from, tegumentary leishmaniasis in Brazil between 2001 and 2020**

| Unit | Total of new cases  (N) | Proportion compared to Brazil  (%) | Cases/year  (mean) | Incidence rates for the entire period  (Total cases/population) x 100.000 inhabitants | Average annual incidence rates  x 100.000 inhabitants | Deaths  (N)* | Mortality rate  (Deaths/population)* x 100.000 inhabitants | Lethality (%)# |
| --- | --- | --- | --- | --- | --- | --- | --- | --- |
| Brazil | 431885 | - | 21594 | 226.41 | 11.32 | 878 | 0.46 | 0.18 |
| Northeast | 129332 | 29.9 | 6467 | 243.65 | 12.18 | 276 | 0.52 | 0.19 |
| North | 182398 | 42.2 | 9120 | 1149.73 | 57.49 | 160 | 1.01 | 0.07 |
| Midwest | 67864 | 15.7 | 3393 | 482.74 | 24.14 | 159 | 1.13 | 0.18 |
| Southeast | 42987 | 10.0 | 2149 | 53.49 | 2.67 | 224 | 0.28 | 0.54 |
| South | 9304 | 2.2 | 465 | 33.97 | 1.70 | 59 | 0.22 | 0.57 |
| Acre | 20790 | 4.8 | 1040 | 2834.13 | 141.71 | 12 | 1.64 | 0.05 |
| Alagoas | 1318 | 0.3 | 66 | 42.24 | 2.11 | 11 | 0.35 | 0.76 |
| Amapá | 12415 | 2.9 | 621 | 1854.30 | 92.71 | 5 | 0.75 | 0.02 |
| Amazonas | 36815 | 8.5 | 1841 | 1056.69 | 52.83 | 11 | 0.32 | 0.02 |
| Bahia | 49180 | 11.4 | 2459 | 350.86 | 17.54 | 89 | 0.63 | 0.17 |
| Ceará | 20088 | 4.7 | 1004 | 237.66 | 11.88 | 73 | 0.86 | 0.31 |
| Federal District | 743 | 0.2 | 37 | 28.91 | 1.45 | 7 | 0.27 | 0.27 |
| Espírito Santo | 3093 | 0.7 | 155 | 88.00 | 4.40 | 8 | 0.23 | 0.36 |
| Goiás | 8616 | 2.0 | 431 | 143.51 | 7.18 | 57 | 0.95 | 0.38 |
| Maranhão | 47038 | 10.9 | 2352 | 715.43 | 35.77 | 54 | 0.82 | 0.11 |
| Mato Grosso | 55576 | 12.9 | 2779 | 1831.10 | 91.55 | 74 | 2.44 | 0.12 |
| Mato Grosso do S. | 2929 | 0.7 | 146 | 119.60 | 5.98 | 21 | 0.86 | 0.61 |
| Minas Gerais | 29108 | 6.7 | 1455 | 148.53 | 7.43 | 136 | 0.69 | 0.45 |
| Pará | 70764 | 16.4 | 3538 | 933.43 | 46.67 | 51 | 0.67 | 0.04 |
| Paraíba | 1181 | 0.3 | 59 | 31.36 | 1.57 | 9 | 0.24 | 0.42 |
| Paraná | 8425 | 2.0 | 421 | 80.66 | 4.03 | 52 | 0.50 | 0.57 |
| Pernambuco | 7872 | 1.8 | 394 | 89.49 | 4.47 | 21 | 0.24 | 0.19 |
| Piauí | 2107 | 0.5 | 105 | 67.57 | 3.38 | 14 | 0.45 | 0.52 |
| Rio de Janeiro | 2292 | 0.5 | 115 | 14.33 | 0.72 | 8 | 0.05 | 0.31 |
| Rio Grande do N. | 257 | 0.1 | 13 | 8.11 | 0.41 | 0 | 0.00 | 0.00 |
| Rio Grande do S. | 171 | 0.0 | 9 | 1.60 | 0.08 | 2 | 0.02 | 1.17 |
| Rondônia | 24058 | 5.6 | 1203 | 1539.80 | 76.99 | 20 | 1.28 | 0.11 |
| Roraima | 7807 | 1.8 | 390 | 1733.04 | 86.65 | 5 | 1.11 | 0.04 |
| Santa Catarina | 708 | 0.2 | 35 | 11.33 | 0.57 | 5 | 0.08 | 0.42 |
| São Paulo | 8494 | 2.0 | 425 | 20.59 | 1.03 | 72 | 0.17 | 1.01 |
| Sergipe | 291 | 0.1 | 14 | 14.07 | 0.70 | 5 | 0.24 | 1.37 |
| Tocantins | 9749 | 2.3 | 487 | 704.69 | 35.23 | 56 | 4.05 | 0.53 |

* Data from the Mortality Information System (SIM)

# Data from the Information System for Notifiable Diseases (SINAN)
