# Supplementary material for: Temporal patterns, spatial risks, and characteristics of tegumentary leishmaniasis in Brazil in the first twenty years of the 21st Century
Source: PLoS Negl Trop Dis. 2023 Jun 7;17(6):e0011405. doi: 10.1371/journal.pntd.0011405 (PMC10281579; doi:10.1371/journal.pntd.0011405)
Supplement: S2 Table — (DOCX) [file pntd.0011405.s002.docx]

| Variables | Brazil | Northeast | North | Midwest | Southeast | South |
| --- | --- | --- | --- | --- | --- | --- |
| Mean / median  (SD / IQR)* | **N** | **N** | **N** | **N** | **N** | **N** |
| Age (years) (2001-2020) | **420412** | **125558** | **176424** | **67292** | **41916** | **9222** |
|  | 33.4 / 31.0  (18.6 / 27.0) | 33.1 / 30.0  (20.3 / 30) | 30.3 / 28.0  (16.1 / 21) | 36.6 / 35.0  (17.4 / 24) | 40.4 / 40.0  (21.0 / 33) | 41.2 / 41.0  (19.4 / 29) |
| Age (years) (2001-2005) | **134470** | **42025** | **52405** | **23165** | **13280** | **3595** |
|  | 32.2 / 29.0 | 32.5 / 29 | 29.2 / 27.0 | 34.7 / 33.0 | 37.2 / 36.0 | 38.1 / 38.0 |
|  | (18.1 / 24.0) | (20.0 / 29.0) | (15.9 / 21) | (16.1 / 22.0) | (20.6 / 32.0) | (19.5 / 29.0) |
|  |  |  |  |  |  |  |
| Age (years) (2006-2010) | **105369** | **33379** | **41707** | **17509** | **10316** | **2458** |
|  | 32.9 / 30.0  (18.6 / 26.0) | 32.2 / 28.0  (20.2 / 28.0) | 29.9 / 27.0  (16.3 / 21.0) | 36.0 / 34.0  (17.0 / 24.0) | 39.6 / 40.0  (20.8 / 32.0) | 40.6 / 41.0  (19.2 / 28.0) |
|  |  |  |  |  |  |  |
| Age (years) (2011-2015) | **101903** | **31453** | **46155** | **14260** | **8087** | **1948** |
|  | 33.7 / 31.0 | 33.5 / 30.0 | 30.6 / 29.0 | 38.2 / 37.0 | 42.2 / 43.0 | 44.3 / 45.0 |
|  | (18.9 / 27.0) | (20.6 / 31.0) | (16.2 / 21.0) | (18.6 / 27.0) | (21.2 / 33.0) | (19.1 / 28.0) |
|  |  |  |  |  |  |  |
| Age (years) (2016-2020) | **78670** | **18701** | **36157** | **12358** | **10233** | **1221** |
|  | 35.7 / 34.0 | 35.4 / 33.0 | 32.0 / 30.0 | 39.4 / 38.0 | 43.6 / 44.0 | 46.5 / 47.0 |
|  | (19.0 / 28.0) | (20.8 / 31.0) | (16.3 / 22.0) | (18.6 / 27.0) | (21.2 / 32.0) | (18.7 / 27.0) |

**Part A - Age profile of new cases of tegumentary Leishmaniasis in Brazil between 2001 and 2020 with stratification by five-year periods**

**S2 Table. Profile and evolution of cases of tegumentary leishmaniasis notified in Brazil and its five major regions between 2001 and 2020.**

***SD = standard deviation; IQR = Interquartile range**

**Part B - Characteristics of new cases of Tegumentary Leishmaniasis in Brazil and its five major regions between 2001 and 2020**

| Variables | Brazil | Northeast | North | Midwest | Southeast | South |
| --- | --- | --- | --- | --- | --- | --- |
| n(%) | **(n = 431885)** | **(n = 129332)** | **(n = 182398)** | **(n = 67864)** | **(n = 42987)** | **(n = 9304)** |
| Sex |  |  |  |  |  |  |
| Masculine | 313103 (72.50) | 80887 (62.54) | 144548 (79.25) | 54464 (80.25) | 26673 (62.05) | 6531 (70.20) |
| Feminine | 118668 (27.48) | 48414 (37.43) | 37803 (20.73) | 13383 (19.72) | 16303 (37.93) | 2765 (29.72) |
| Missing data | 114 (0.03) | 31 (0.02) | 47 (0.03) | 17 (0.03) | 11 (0.03) | 8 (0.09) |
| Age |  |  |  |  |  |  |
| <1 year | 3975 (0.92) | 1260 (0.97) | 1828 (1.00) | 531 (0.78) | 310 (0.72) | 46 (0.49) |
| 1-4 years | 10439 (2.42) | 4261 (3.29) | 4320 (2.37) | 848 (1.25) | 880 (2.05) | 130 (1.40) |
| 5-9 years | 17782 (4.12) | 7547 (5.84) | 6959 (3.82) | 1309 (1.93) | 1660 (3.86) | 307 (3.30) |
| 10-19 years | 73997 (17.13) | 24755 (19.16) | 34222 (18.76) | 8532 (12.57) | 5551 (12.91) | 917 (9.86) |
| 20-34 years | 135046 (31.27) | 35256 (27.26) | 67192 (36.84) | 21732 (32.02) | 8772 (20.41) | 2094 (22.51) |
| 35 - 49 years | 95174 (22.04) | 24783 (19.16) | 39199 (21.49) | 18697 (27.55) | 9950 (23.15) | 2545 (27.35) |
| ≥ 50 years | 83999 (19.45) | 27676 (21.40) | 22704 (12.45) | 15643 (23.05) | 14793 (34.41) | 3183 (34.21) |
| Missing data | 11473 (2.66) | 3774 (2.92) | 5974 (3.28) | 572 (0.84) | 1071 (2.49) | 82 (0.88) |
| Ethnicity/skin color | |  |  |  |  |  |
| White | 93262 (21.59) | 15538 (12.01) | 28974 (15.89) | 25555 (37.66) | 16921 (39.36) | 6274 (67.43) |
| Non White | 284242 (65.81) | 92938 (71.86) | 132046 (72.39) | 37350 (55.04) | 20426 (47.42) | 1482 (15.93) |
| Missing data | 54381 (12.59) | 20856 (16.13) | 21378 (11.72) | 4959 (7.31) | 5640 (13.12) | 1548 (16.64) |
| Location |  |  |  |  |  |  |
| Urban | 186859 (43.27) | 41040 (31.73) | 81973 (44.94) | 38076 (56.11) | 20296 (47.21) | 5474 (58.83) |
| Rural | 227325 (52.64) | 84036 (64.98) | 92697 (50.82) | 26964 (39.73) | 20300 (47.22) | 3328 (35.77) |
| Periurban | 3509 (0.81) | 874 (0.68) | 1096 (0.60) | 743 (1.09) | 683 (1.59) | 113 (1.21) |
| Missing data | 14192 (3.29) | 3382 (2.61) | 6632 (3.64) | 2081 (3.07) | 1+708 (3.97) | 389 (4.18) |
| Education | |  |  |  |  |  |
| No formal instruction | 14536 (3.37) | 7084 (5.48) | 4150 (2.28) | 1798 (2.65) | 1256 (2.92) | 248 (2.67) |
| Unitl elementary school | 141564 (32.78) | 42417 (32.80) | 62557 (34.30) | 22287 (32.84) | 11596 (26.77) | 2797 (30.06) |
| Until high school | 35412 (8.20) | 7532 (5.82) | 16078 (8.81) | 7339 (10.81) | 3535 (8.22) | 928 (9.97) |
| Up to higher education | 5843 (1.35) | 901 (0.70) | 2201 (1.21) | 1587 (2.34) | 909 (2.11) | 245 (2.63) |
| Missing data /N.A. | 234530 (54.30) | 71398 (55.21) | 97412 (53.41) | 34853 (51.36) | 25781 (59.97) | 5086 (54.66) |
| Disease confirmation |  |  |  |  |  |  |
| Clinical-epidemiological | 75707 (17.53) | 43520 (33.65) | 14925 (8.18) | 7902 (11.64) | 7849 (18.26) | 1511 (16.24) |
| Laboratory | 350724 (81.21) | 83731 (64.74) | 165894 (90.95) | 59007 (86.95) | 34496 (80.25) | 7596 (81.64) |
| Missing data | 5454 (1.26) | 2081 (1.61) | 1579 (0.87) | 955 (1.41) | 642 (1.49) | 197 (2.12) |
| Co-infection with HIV |  |  |  |  |  |  |
| Yes | 2245 (0.52) | 507 (0.39) | 1131 (0.62) | 314 (0.46) | 234 (0.54) | 59 (0.63) |
| No | 136933 (31.71) | 38815 (35.84) | 53157 (29.14) | 24321 (35.84) | 16976 (39.49) | 3664 (39.38) |
| Missing data | 292707 (67.77) | 90010 (69.60) | 128110 (70.24) | 43229 (63.70) | 25777 (59.96) | 5581 (59.98) |
| First treatment |  |  |  |  |  |  |
| Amphotericin | 4414 (1.02) | 937 (0.32) | 947 (0.52) | 725 (1.07) | 1632 (3.80) | 173 (1.86) |
| Antimoniate | 374796 (86.78) | 115374 (89.21) | 156625 (85.87) | 61393 (90.46) | 33578 (78.11) | 7826 (84.11) |
| Not used | 6146 (1.42) | 1833 (1.42) | 2037 (1.12) | 891 (1.31) | 1128 (2.62) | 257 (2.76) |
| Others | 17527(4.06) | 4648 (3.59) | 7867 (4.31) | 2249 (3.31) | 2366 (5.50) | 397 (4.27) |
| Pentamidine | 5868 (1.36) | 279 (0.22) | 4933 (2.70) | 452 (0.67) | 161 (0.37) | 43 (0.46) |
| Missing data | 23134 (5.36) | 6261 (4.84) | 9989 (5.48) | 2154 (3.17) | 4122 (9.49) | 608 (6.53) |
| Clinical form |  |  |  |  |  |  |
| Cutaneous | 407737 (94.41) | 124965 (96.62) | 172709 (94.69) | 63050 (92.91) | 38822 (90.31) | 8191 (88.04) |
| Mucocutaneous | 23749 (5.50) | 4202 (3.25) | 9589 (5.26) | 4777 (7.04) | 4091 (9.52) | 1090 (11.72) |
| Missing data | 399 (0.9) | 165 (0.13) | 100 (0.05) | 37 (0.05) | 74 (0.17) | 23 (0.25) |
| Evolution |  |  |  |  |  |  |
| Cure | 319487 (73.98) | 95713 (74.01) | 132407 (72.59) | 52409 (77.23) | 31860 (74.12) | 7098 (76.29) |
| Death | 779 (0.18) | 243 (0.19) | 128 (0.07) | 121 (0.18) | 234 (0.54) | 53 (0.57) |
| Outher | 24306 (5.63) | 4937 (3.82) | 13059 (7.16) | 2709 (3.99) | 2976 (6.92) | 625 (6.72) |
| Missing data | 87313 (20.22) | 28439 (21.99) | 36804 (20.18) | 12625 (18.60) | 7917 (18.42) | 1528 (16.42) |

**Part C - Temporal evolution of the characteristics of new cases of tegumentary Leishmaniasis between 2001 and 2020**

| Variables | 2001 - 2005 | 2006-2010 | 2011-2015 | 2016-2020 |
| --- | --- | --- | --- | --- |
| n(%) | **(n = 140528)** | **(n = 108042 )** | **(n = 103626 )** | **(n = 79678)** |
| Sex |  |  |  |  |
| Masculine | 39317 (27.98) | 29743 (27.53) | 28415 (27.42) | 21191 (26.60) |
| Feminine | 101124 (71.96) | 78291 (72.46) | 75196 (72.56) | 58483 (73.40) |
| Missing data | 87 (0.06) | 8 (0.01) | 15 (0.01) | 4 (0.01) |
| Idade |  |  |  |  |
| <1 year | 881 (0.63) | 1071 (0.99) | 1250 (1.21) | 773 (0.97) |
| 1-4 years | 3475 (2.47) | 2868 (2.65) | 2424 (2.34) | 1672 (2.10) |
| 5-9 years | 6064 (4.32) | 4795 (4.44) | 4277 (4.13) | 2646 (3.32) |
| 10-19 years | 25314 (18.01) | 18781 (17.38) | 17896 (17.27) | 12005 (15.07) |
| 20-34 years  35-49 years | 45056 (32.06)  30025 (21.37) | 34391 (31.83)  23297 (21.56) | 31864 (30.75)  22887 (22.09) | 23735 (29.79)  18960 (23.80) |
| ≥ 50 years | 23644 (16.83) | 20166 (18.66) | 21305 (20.56) | 18879 (23.69) |
| Missing data | 6069 (4.3) | 2673 (2.5) | 1723 (1.7) | 1008 (1.27) |
| Ethnicity/skin color | |  |  |  |
| White | 32396 (23.05) | 26017 (24.08) | 19526 (18.84) | 15323 (19.23) |
| Non White | 66137 (47.06) | 75565 (69.94) | 80658 (77.84) | 61882 (77.67) |
| Missing data | 41995 (29.88) | 6460 (5.98) | 3442 (3.32) | 2473 (3.10) |
| Location |  |  |  |  |
| Urban | 63708 (45.33) | 45919 (42.50) | 43351 (41.83) | 33876 (42.52) |
| Rural | 69995 (49.81) | 57889 (53.58) | 56528 (54.55) | 42907 (53.85) |
| Periurban | 1337 (0.95) | 894 (0.83) | 703 (0.68) | 575 (0.72) |
| Missing data | 5488 (3.91) | 3340 (3.09) | 3044 (2.94) | 2320 (2.91) |
| Education | |  |  |  |
| No formal instruction | 0 (0.00) | 5204 (4.82) | 5915 (5.71) | 3417 (4.29) |
| Unitl elementary school | 0 (0.00) | 50036 (46.31) | 53681 (51.80) | 37847 (47.50) |
| Until high school | 0 (0.00) | 7565 (7.00) | 13296 (12.83) | 14551 (18.26) |
| Up to higher education | 0 (0.00) | 1240 (1.15) | 2213 (2.14) | 2390 (3.00) |
| Missing data /N.A. | 140528 (100.00) | 43997 (40.72) | 28521 (27.52) | 21473 (26.95) |
| Disease confirmation |  |  |  |  |
| Clinical-epidemiological | 27068 (19.26) | 17016 (15.75) | 16662 (16.08) | 14956 (18.77) |
| Laboratory | 108661 (77.32) | 90396 (83.67) | 86964 (83.92) | 64697 (81.20) |
| Missing data | 4799 (3.41) | 630 (0.58) | 0 (0.00) | 25 (0.03) |
| Co-infection with HIV |  |  |  |  |
| Yes | 0 (0.00) | 840 (0.78) | 717 (0.69) | 688 (0.86) |
| No | 0 (0.00) | 43173 (39.96) | 46107 (44.49) | 47653 (59.81) |
| Missing data | 140528 (100.00) | 64029 (59.26) | 56802 (54.81) | 31337 (39.33) |
| First treatment |  |  |  |  |
| Amphotericin | 517 (0.37) | 693 (0.64) | 1169 (1.13) | 2035 (2.55) |
| Antimoniate | 118468 (84.30) | 97797 (90.52) | 92057 (88.84) | 66465 (83.42) |
| Not used | 2703 (1.92) | 1359 (1.26) | 1008 (0.97) | 1076 (1.35) |
| Others | 7175 (5.11) | 3644 (3.37) | 3494 (3.37) | 3213 (4.03) |
| Pentamidine | 1921 (1.37) | 795 (0.74) | 1292 (1.25) | 1859 (2.33) |
| Missing data | 9744 (6.93) | 3754 (3.47) | 4606 (4.44) | 5030 (6.31) |
| Clinical form |  |  |  |  |
| Cutaneous | 132086 (93.99) | 101830 (94.25) | 98111 (94.68) | 75701 (95.01) |
| Mucosal | 8053 (5.73) | 6212 (5.75) | 5515 (5.32) | 3968 (4.98) |
| Missing data | 389 (0.28) | 0 (0.00) | 0 (0.00) | 9 (0.01) |
