# Supplementary material for: Temporal patterns, spatial risks, and characteristics of tegumentary leishmaniasis in Brazil in the first twenty years of the 21st Century
Source: PLoS Negl Trop Dis. 2023 Jun 7;17(6):e0011405. doi: 10.1371/journal.pntd.0011405 (PMC10281579; doi:10.1371/journal.pntd.0011405)
Supplement: S1 Fig — (DOCX) [file pntd.0011405.s003.docx]

**S1 Fig. Joinpoint analysis (left) and generalized additive models (right) of cases of tegumentary leishmaniasis notified in the 27 federative units of Brazil between 2001 and 2020.**

- **Acre**

**
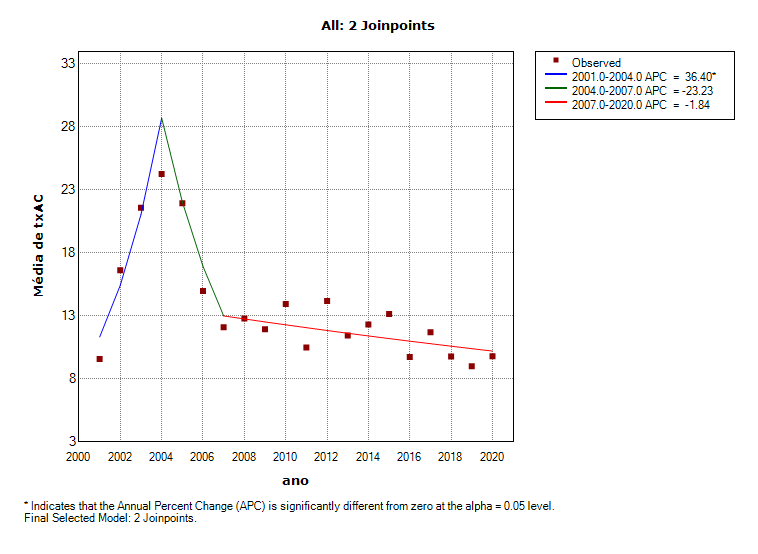

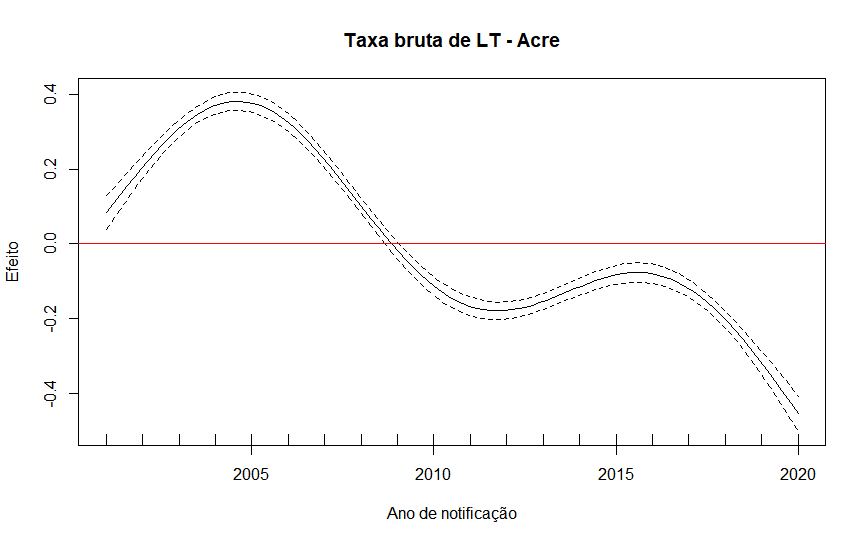
**

- **Alagoas**

**
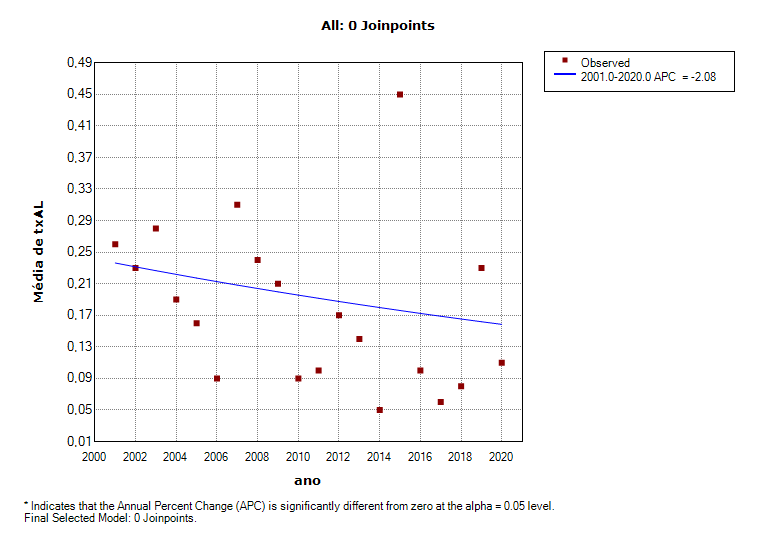

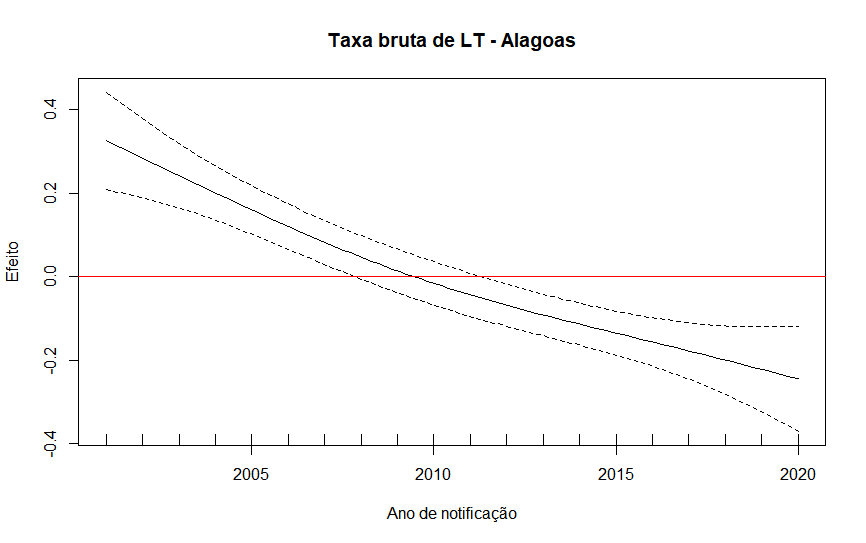
**

- **Amazonas**

**
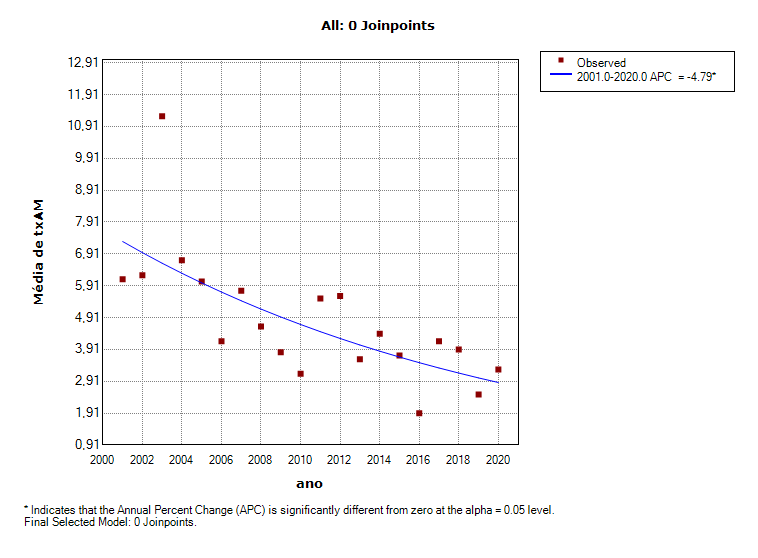

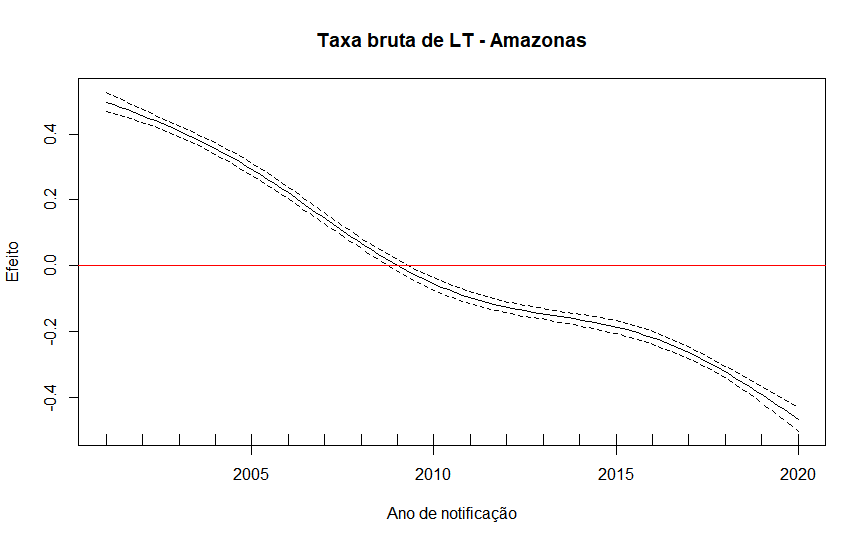
**

- **Amapá**

**
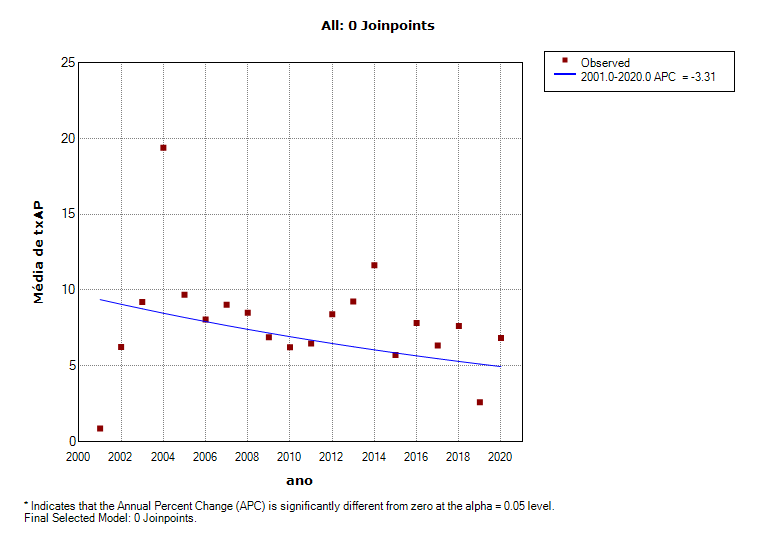

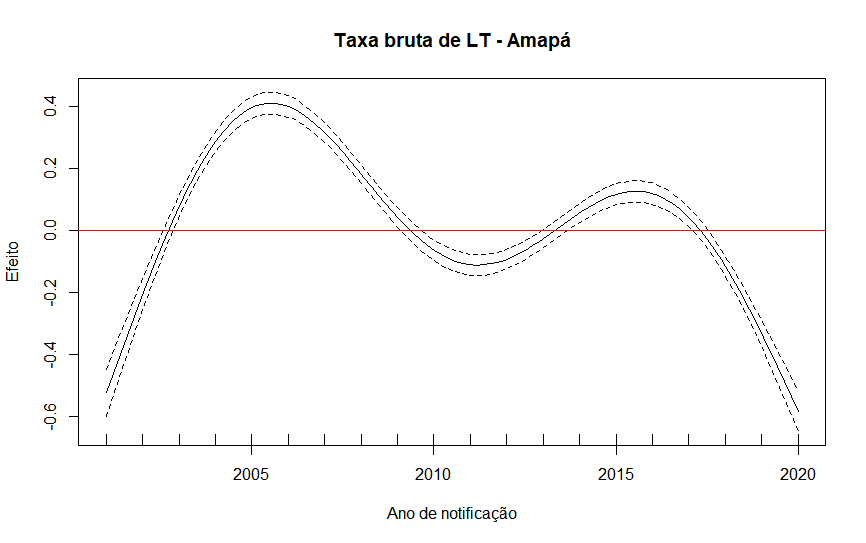
**

- **Bahia**

**
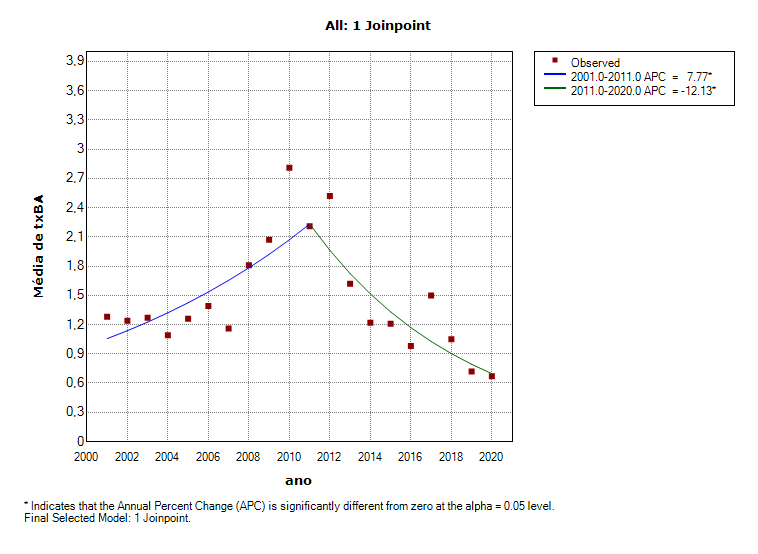

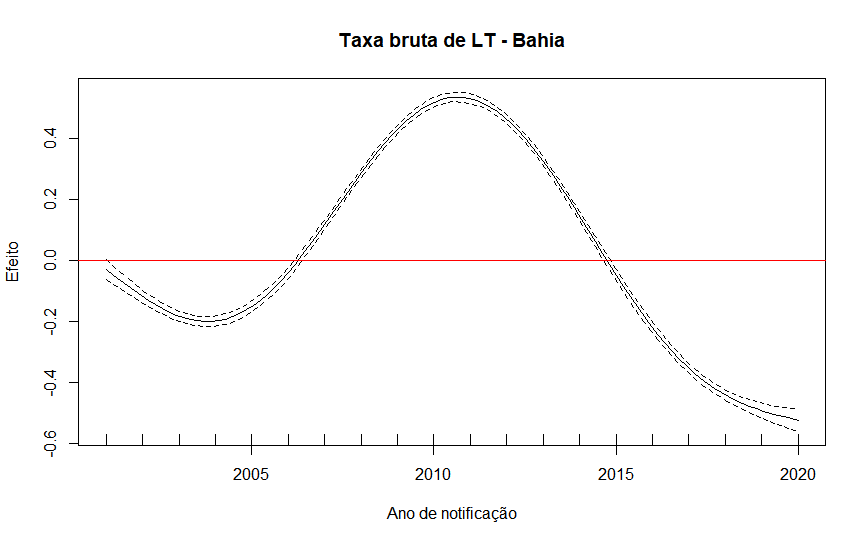
**

- **Ceará**


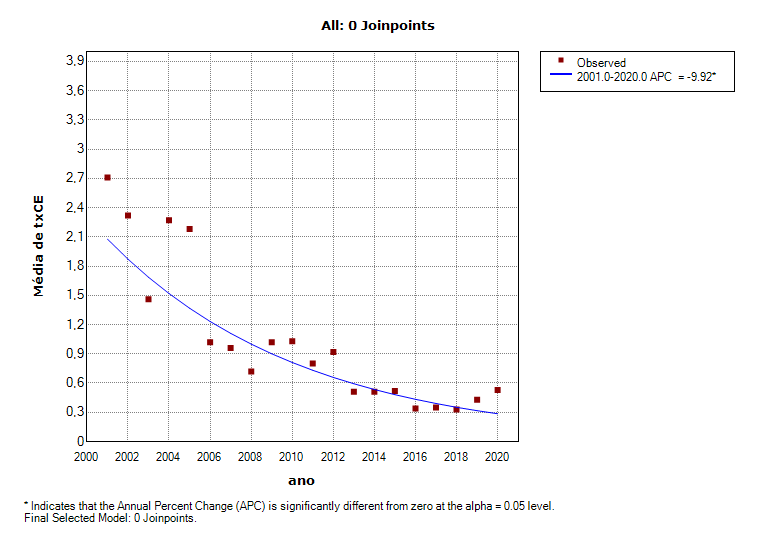

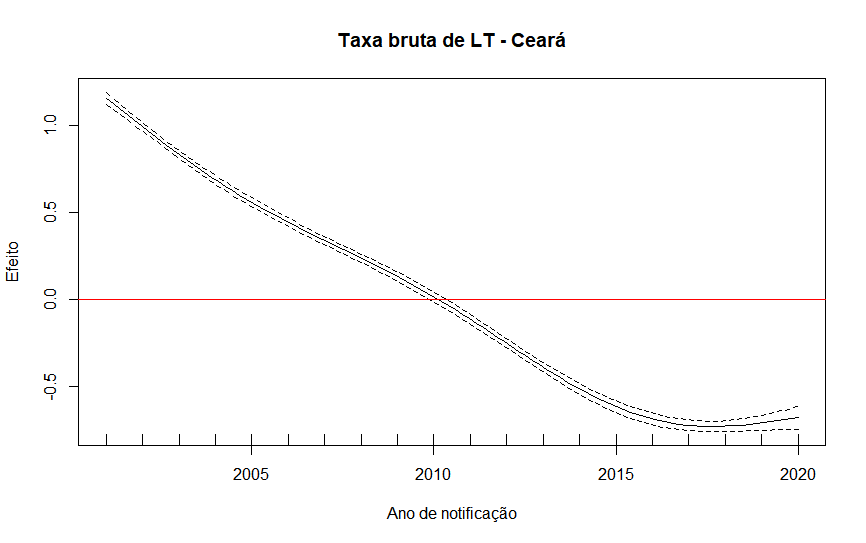


- **Distrito Federal**

**
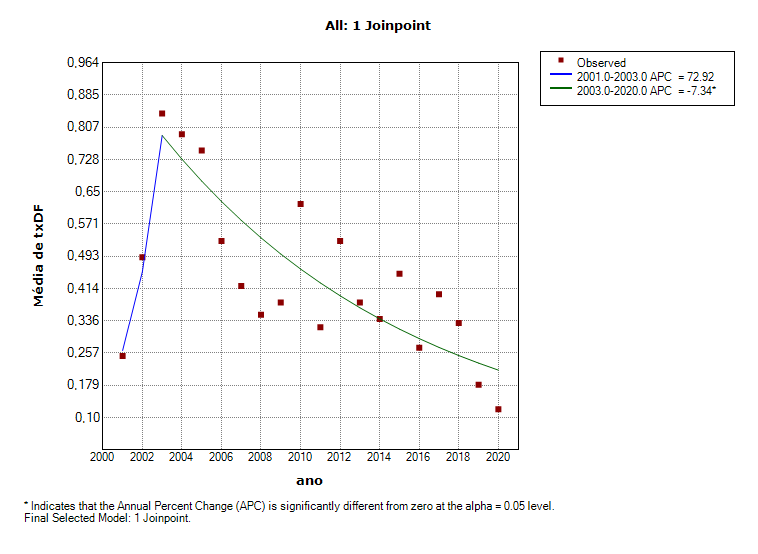

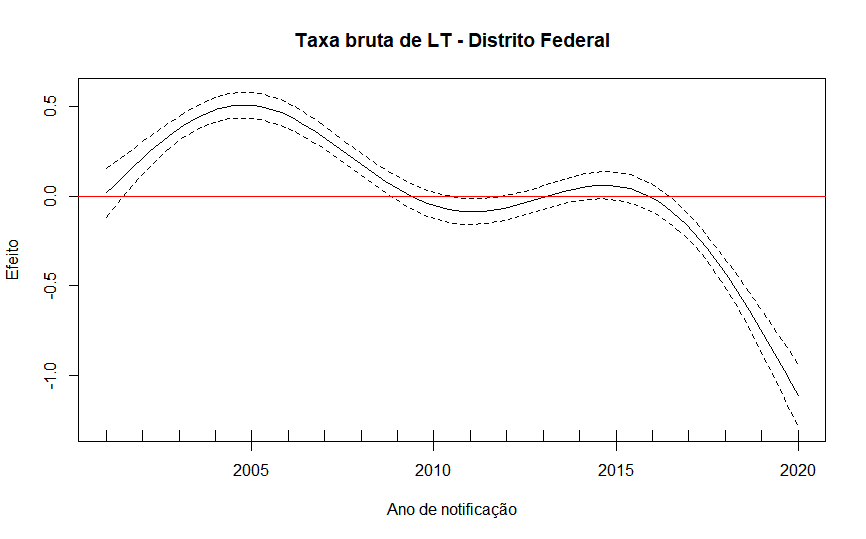
**

- **Espírito Santo**

**
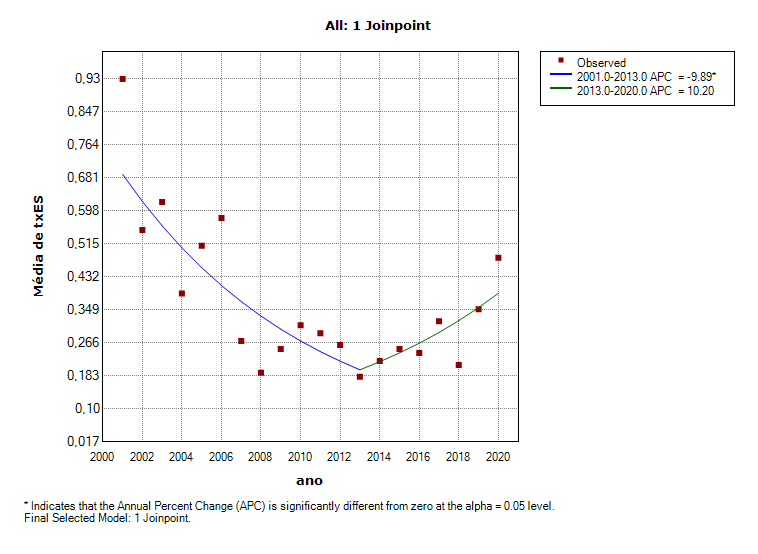

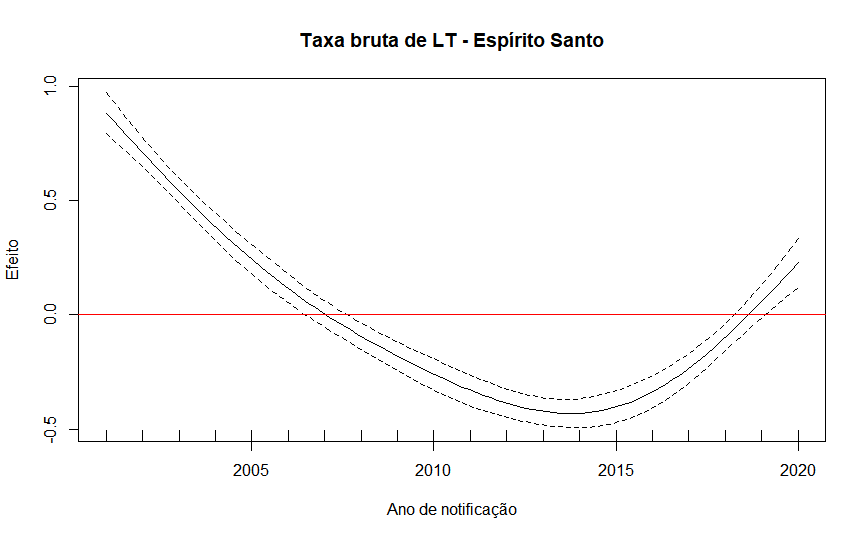
**

- **Goiás**

**
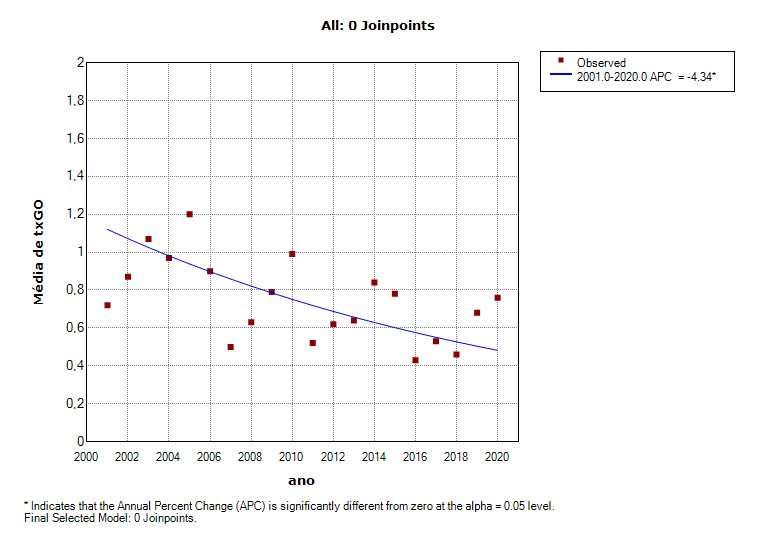

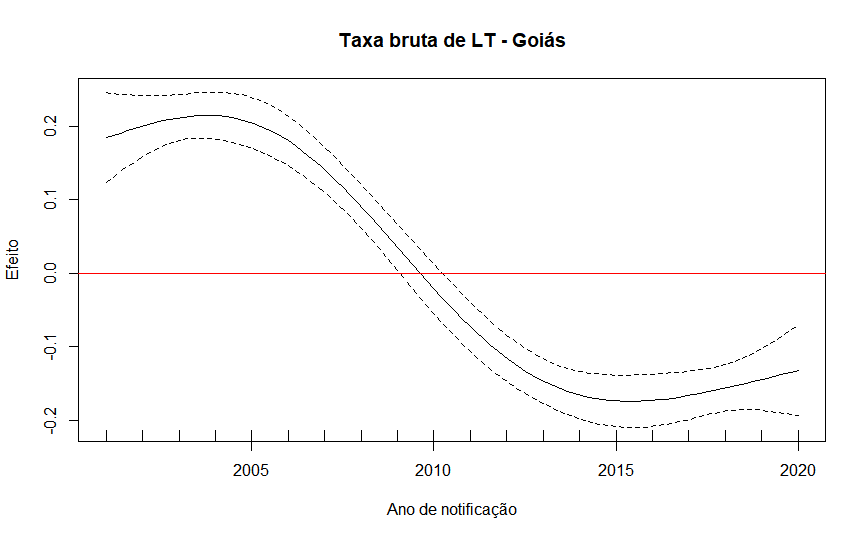
**

- **Maranhão**

**
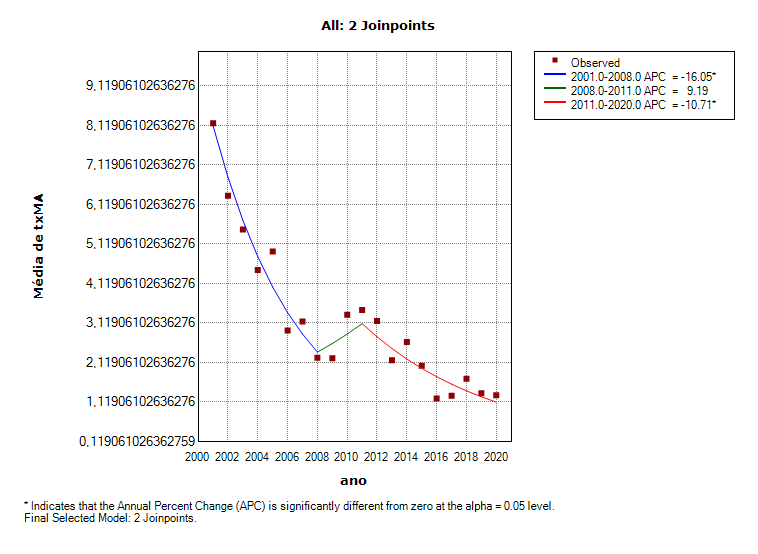

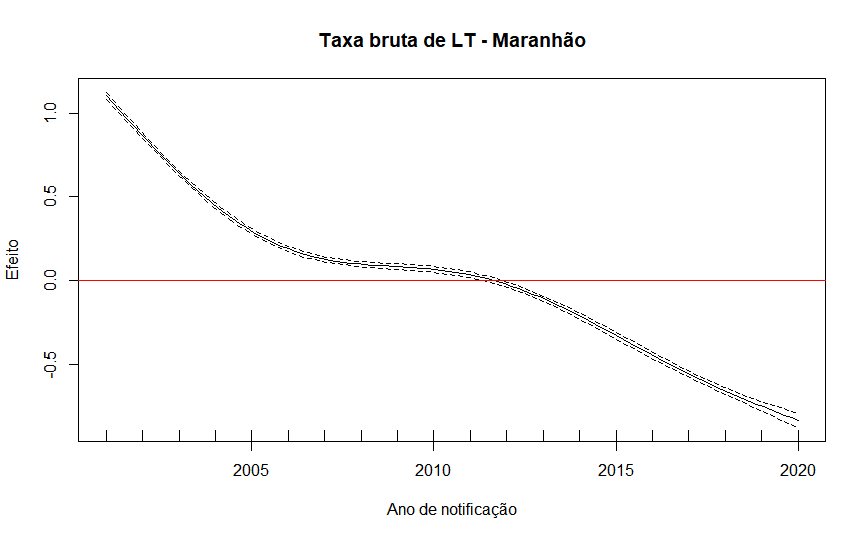
**

- **Minas Gerais**

**
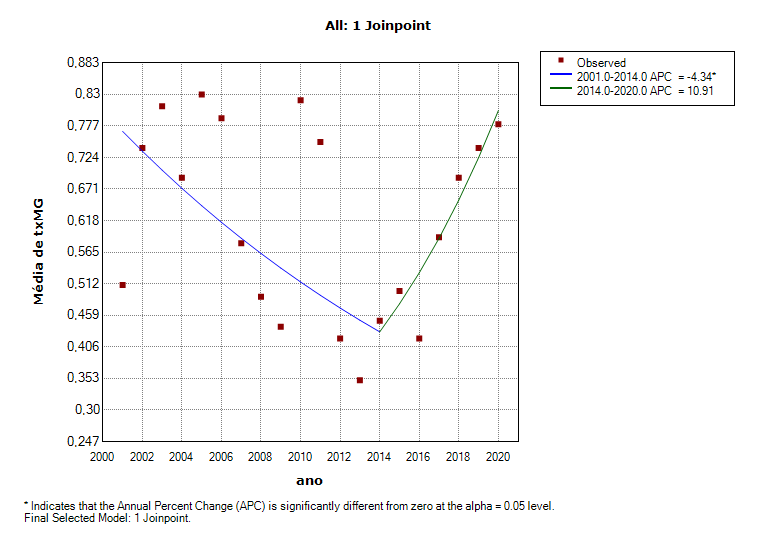

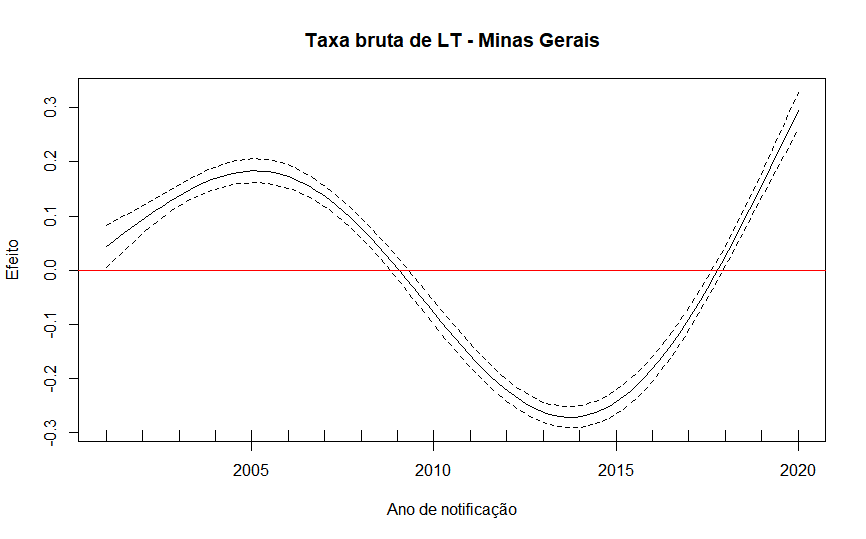
**

- **Mato Grosso do Sul**

**
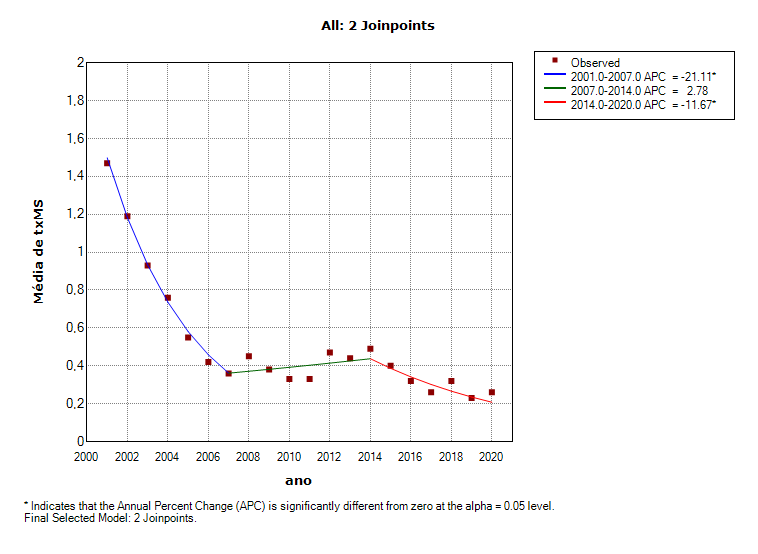

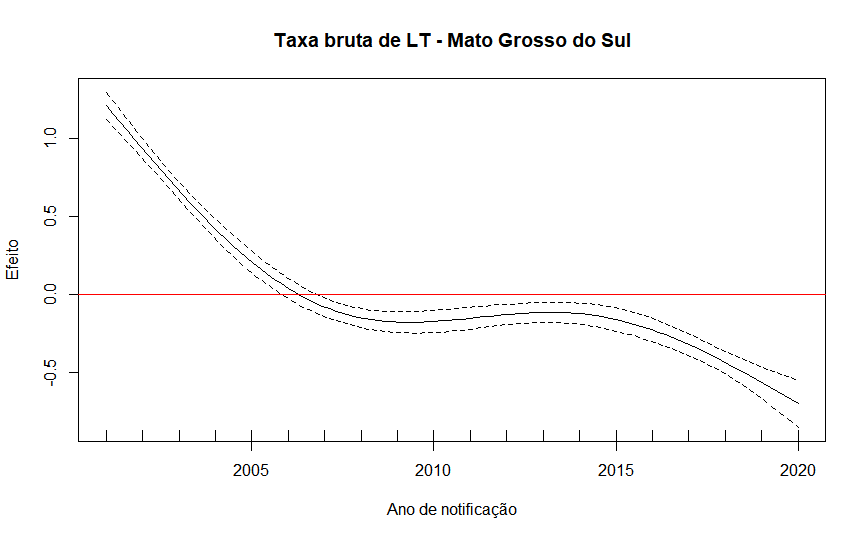
**

- **Mato Grosso**

**
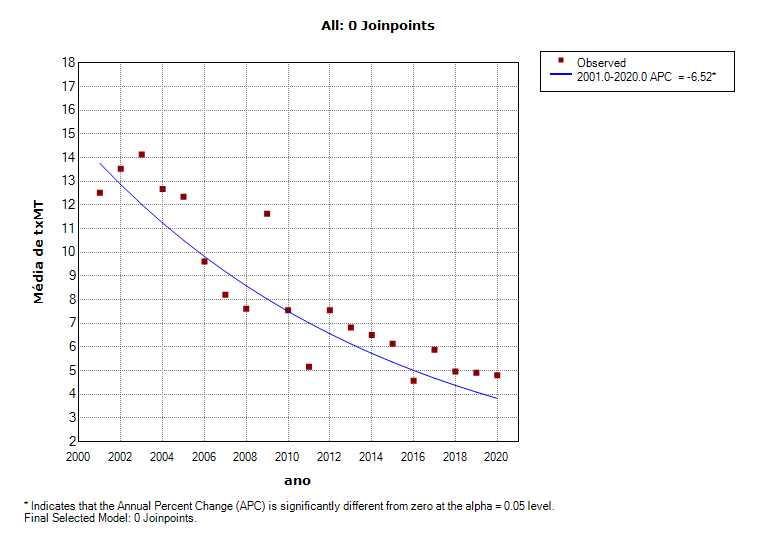

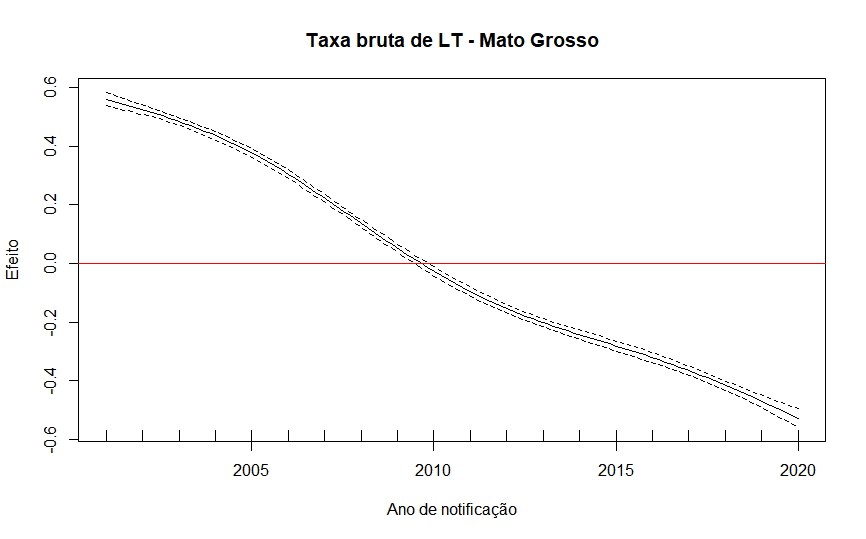
**

- **Pará**

**
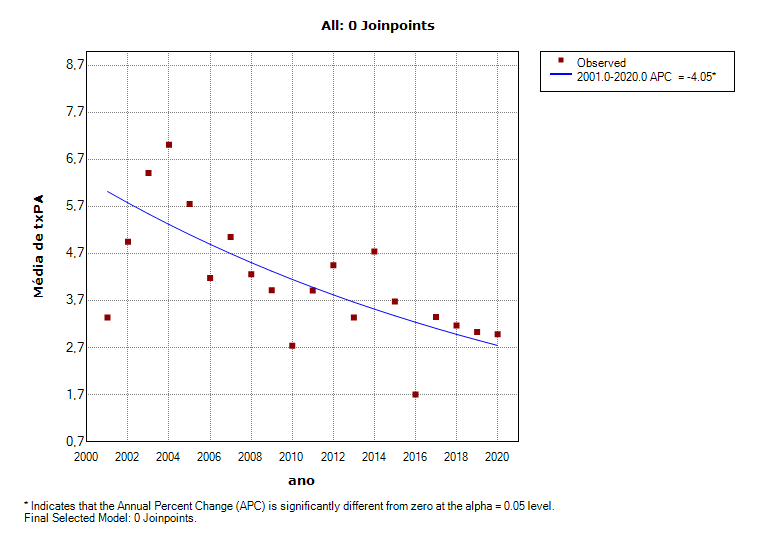

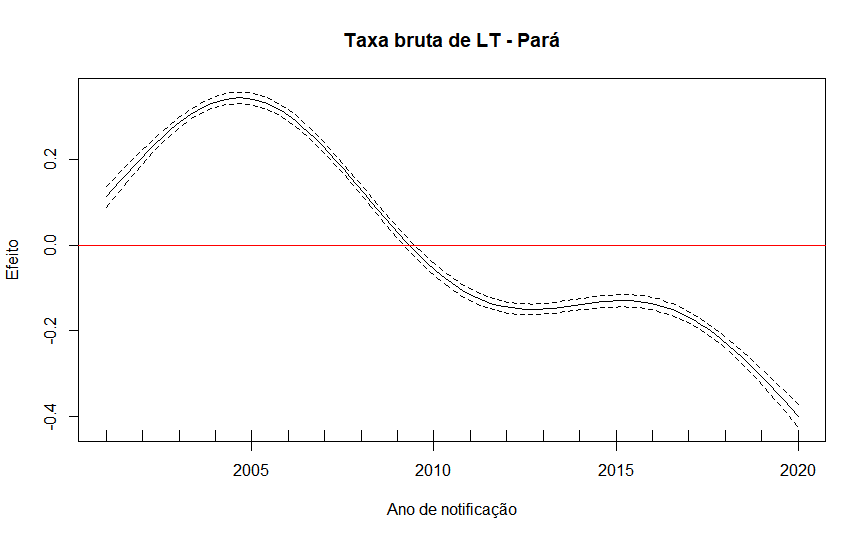
**

- **Paraíba**

**
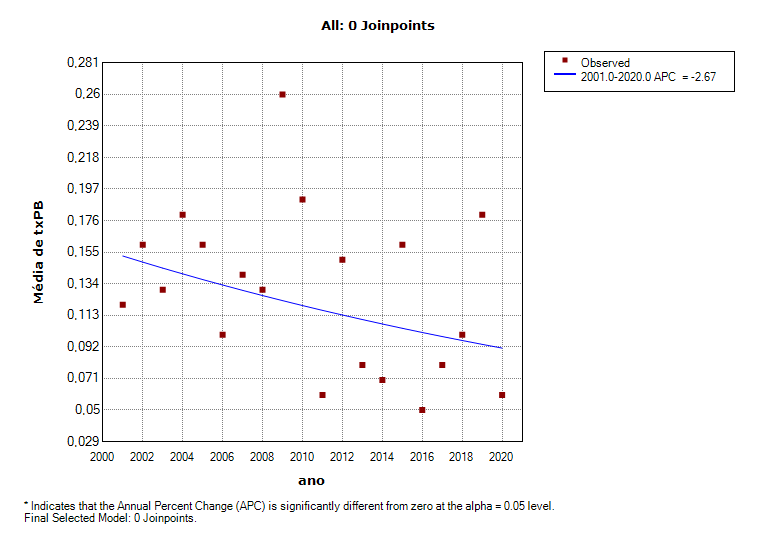

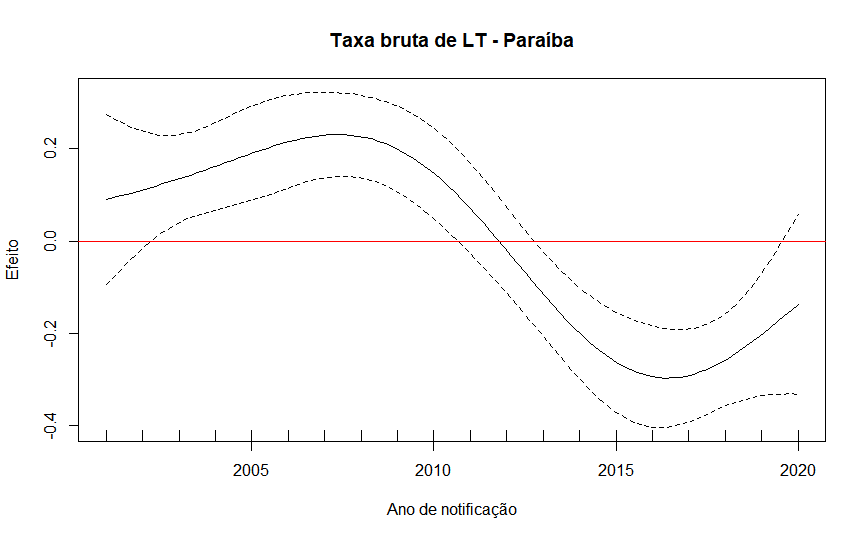
**

- **Pernambuco**

**
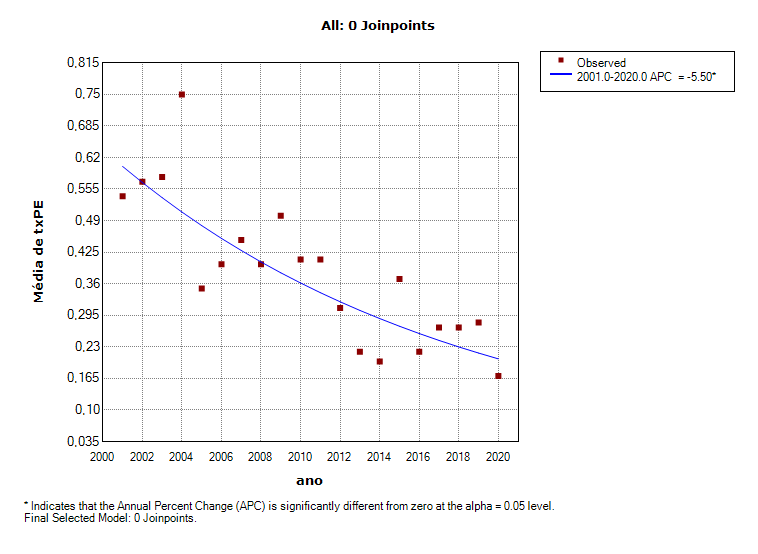

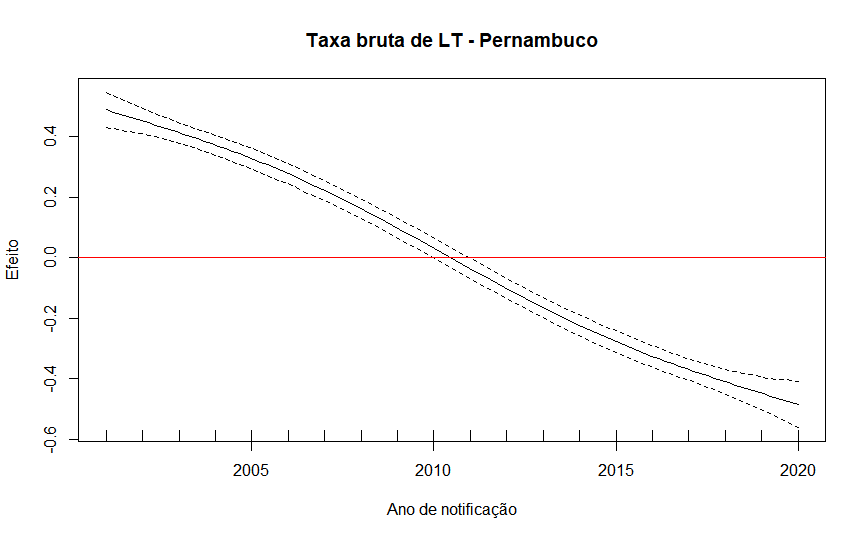
**

- **Piauí**

**
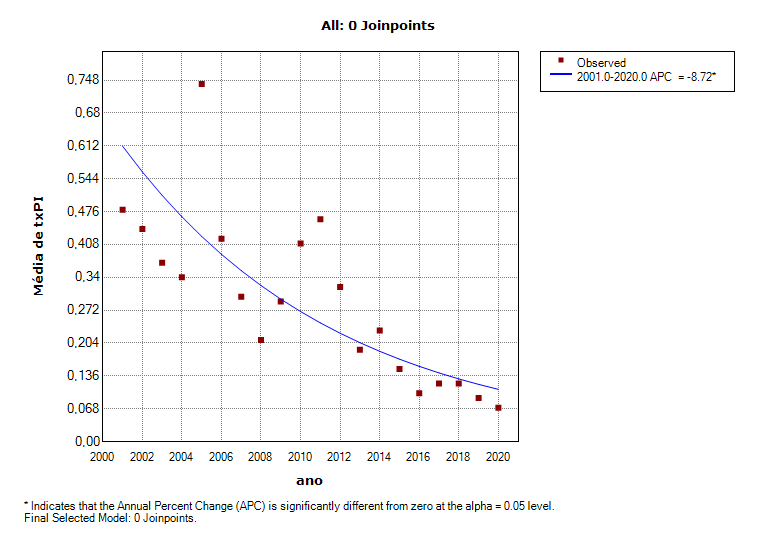

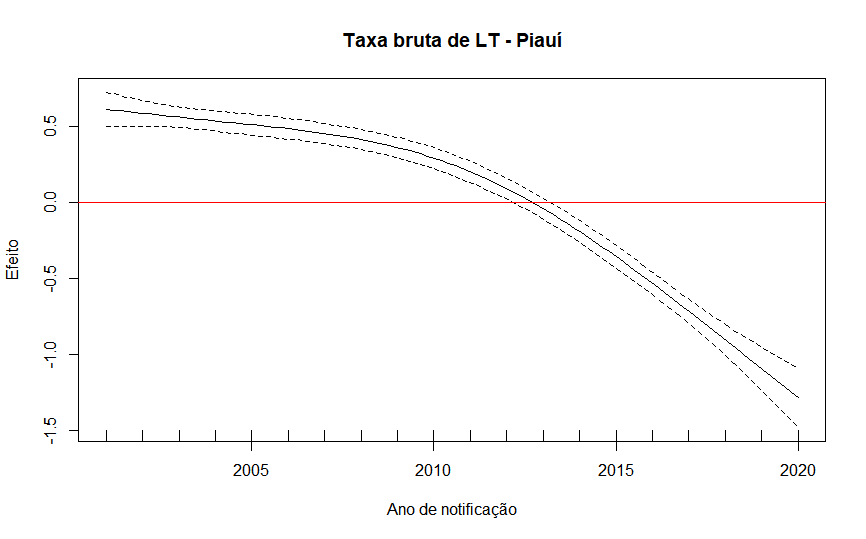
**

- **Paraná**

**
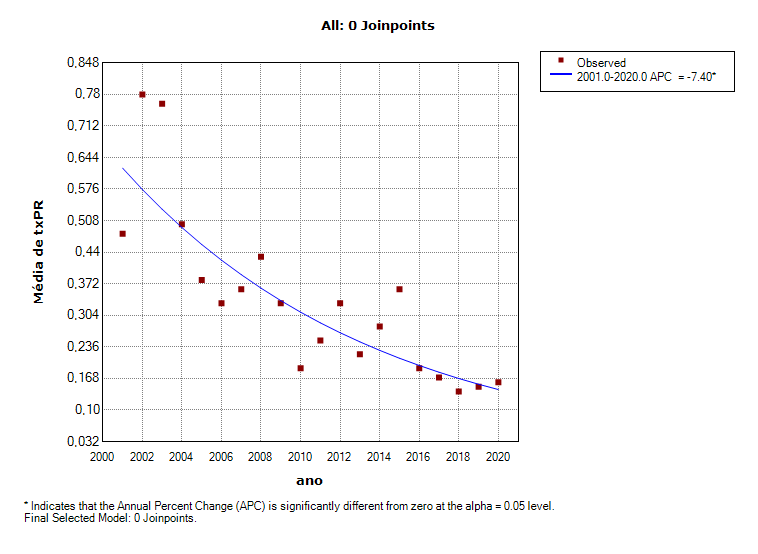

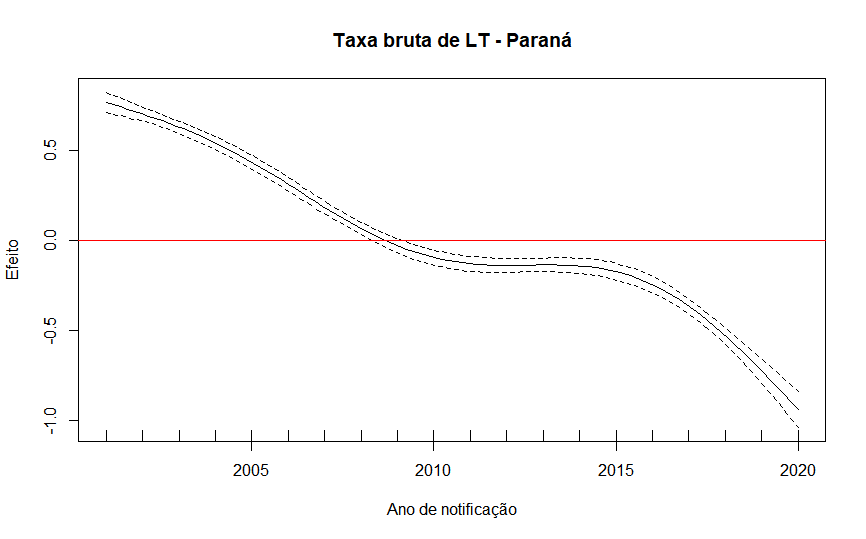
**

- **Rio de Janeiro**

**
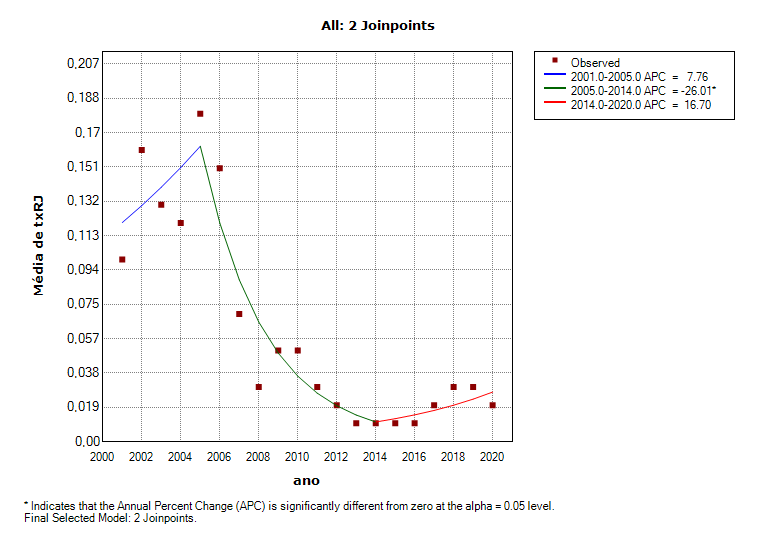

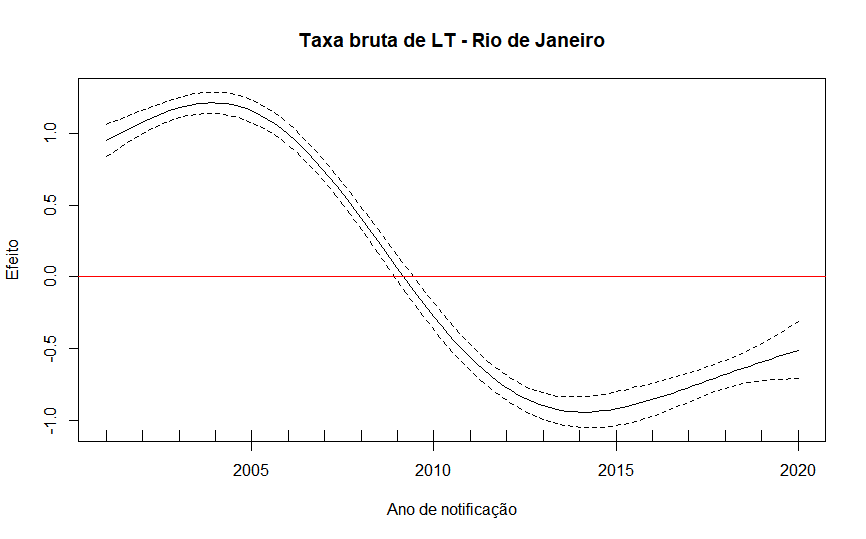
**

- **Rio Grande do Norte**

Joinpoint not adjusted due to monthly incidence = 0 **
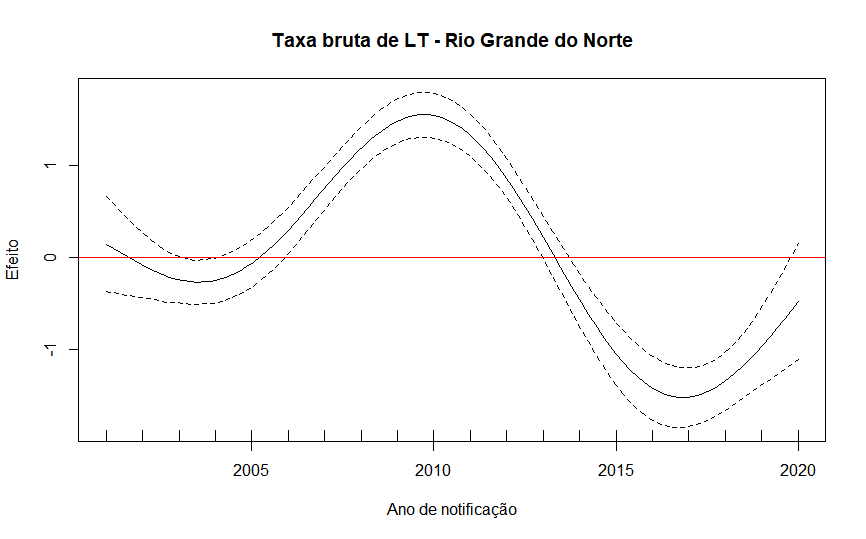
**

- **Rondônia**

**
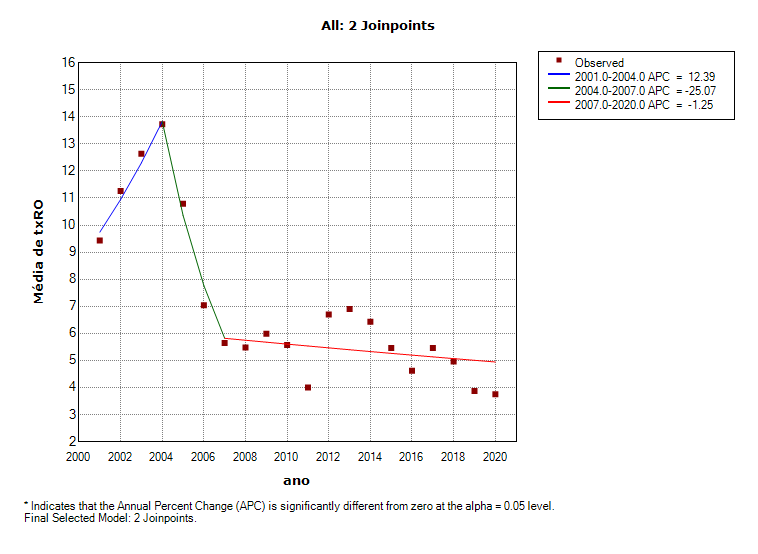

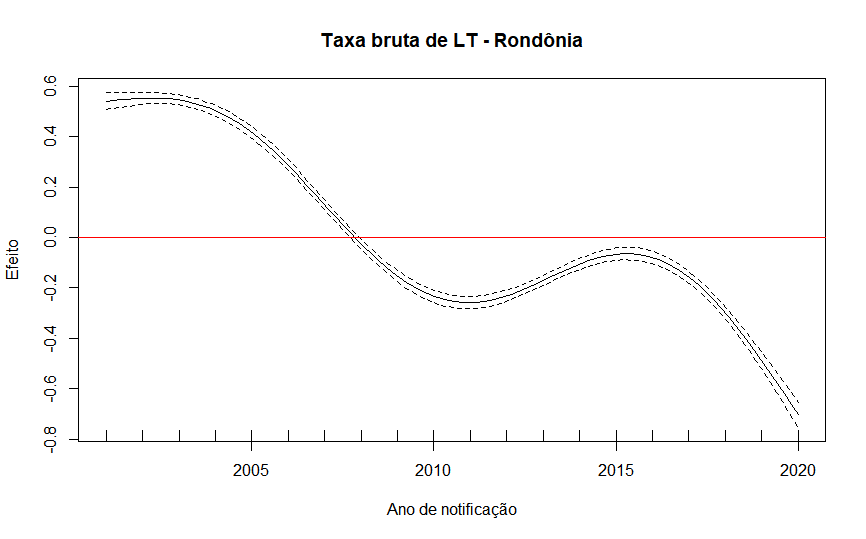
**

- **Roraima**

**
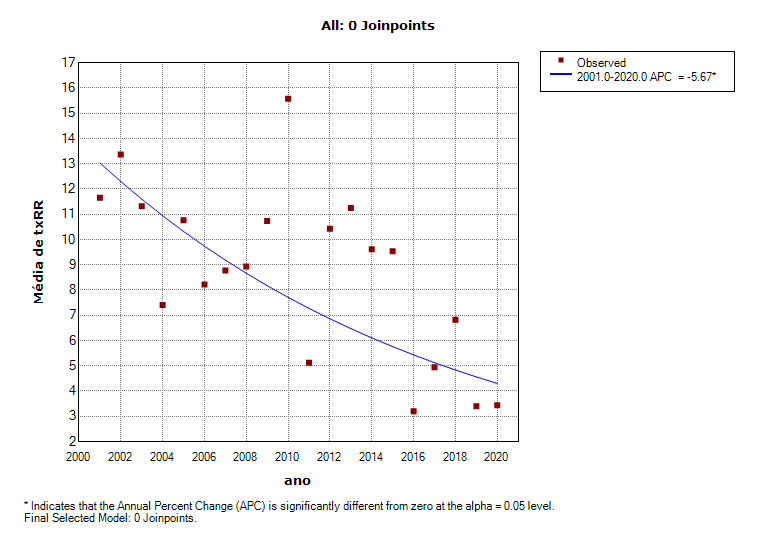

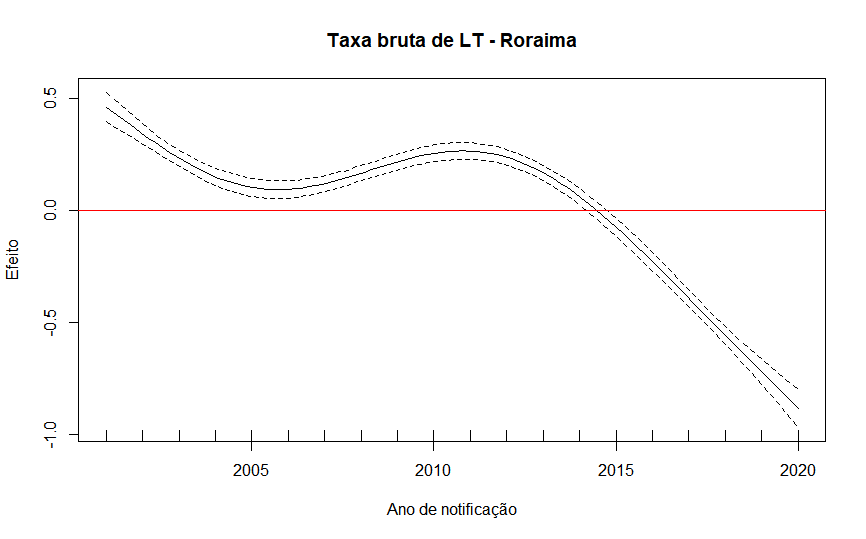
**

- **Rio Grande do Sul**

Joinpoint not adjusted due to monthly incidence = 0 **
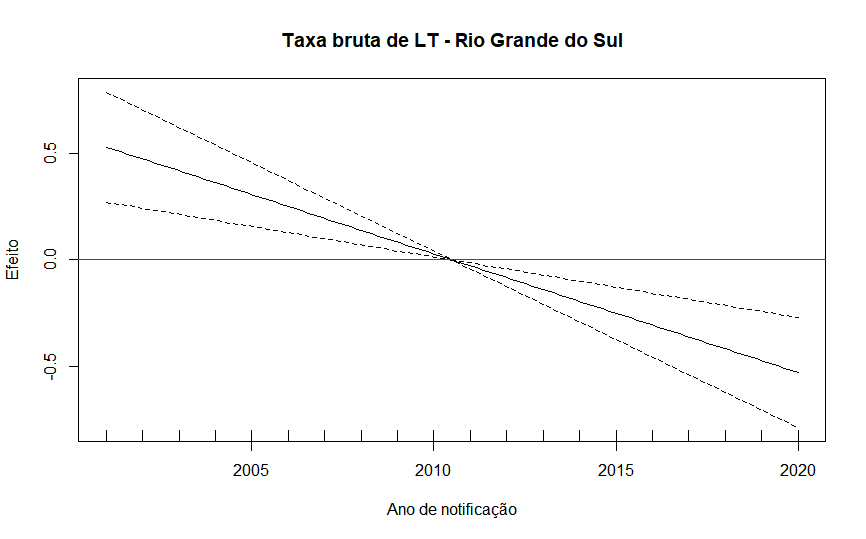
**

- **Santa Catarina**

**
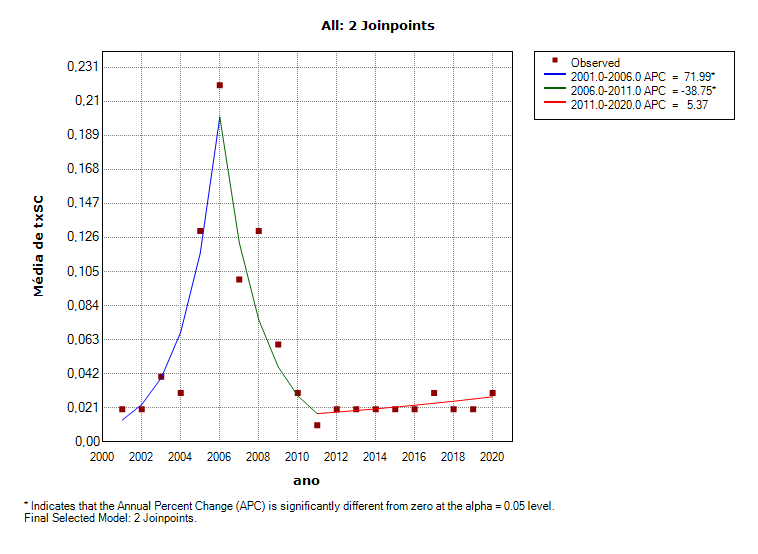

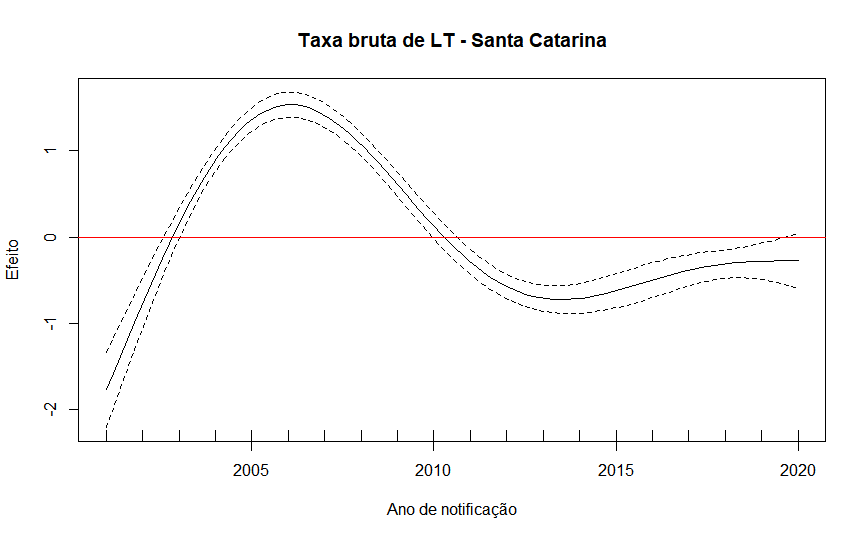
**

- **Sergipe**

**
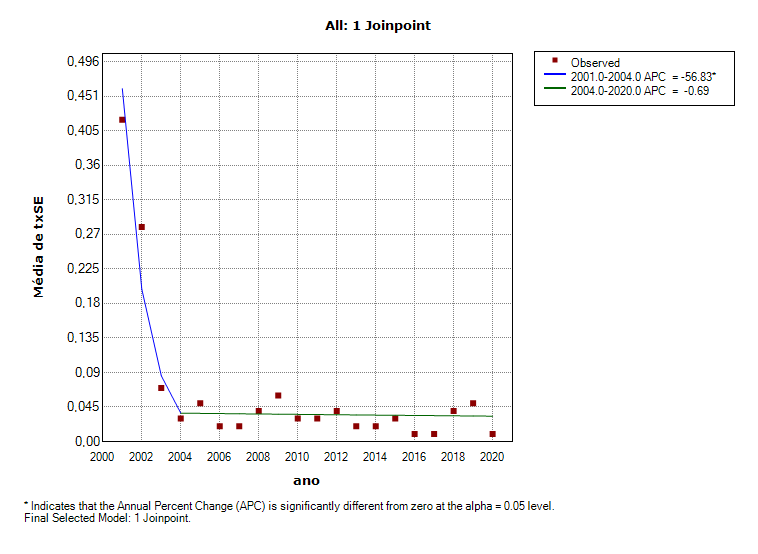

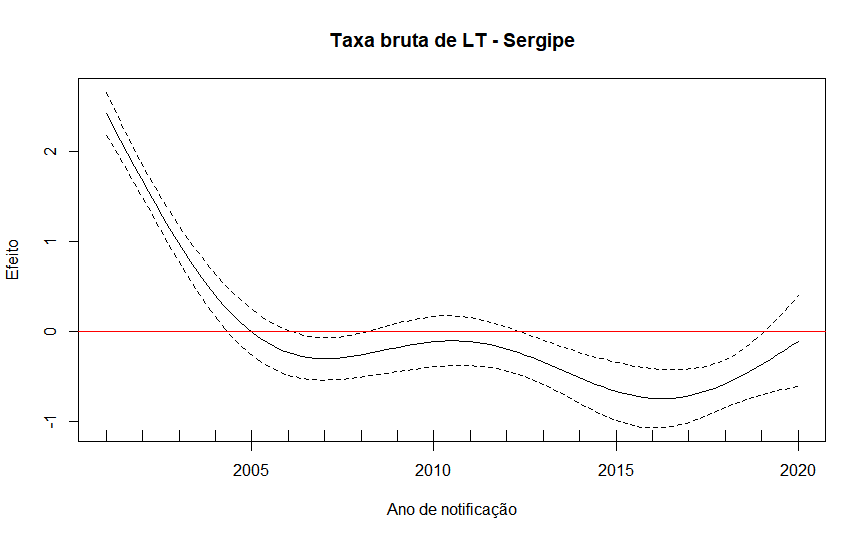
**

- **São Paulo**

**
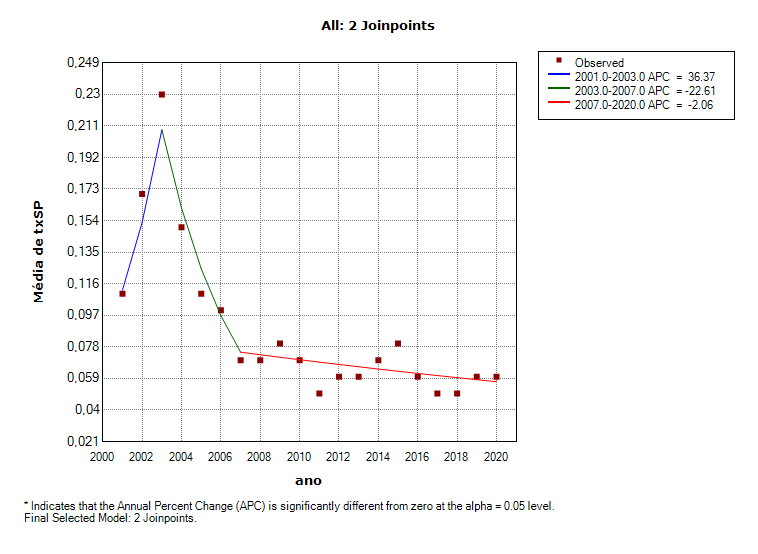

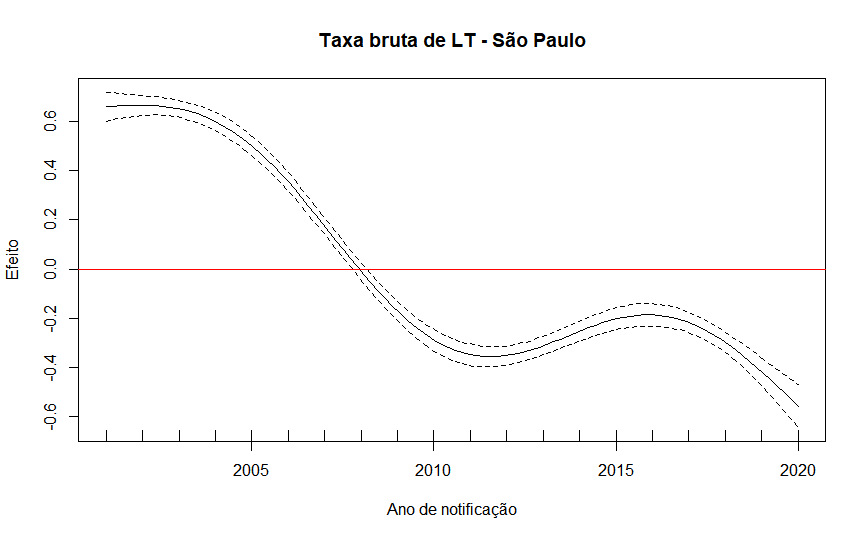
**

- **Tocantins**

**
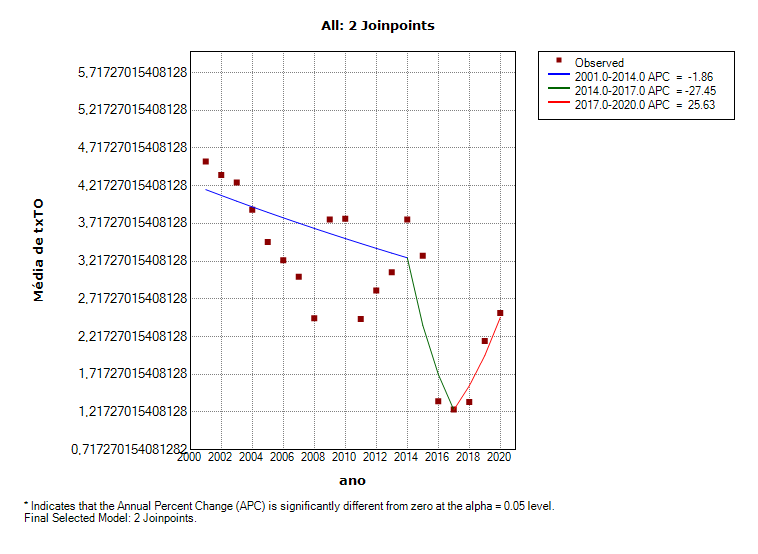

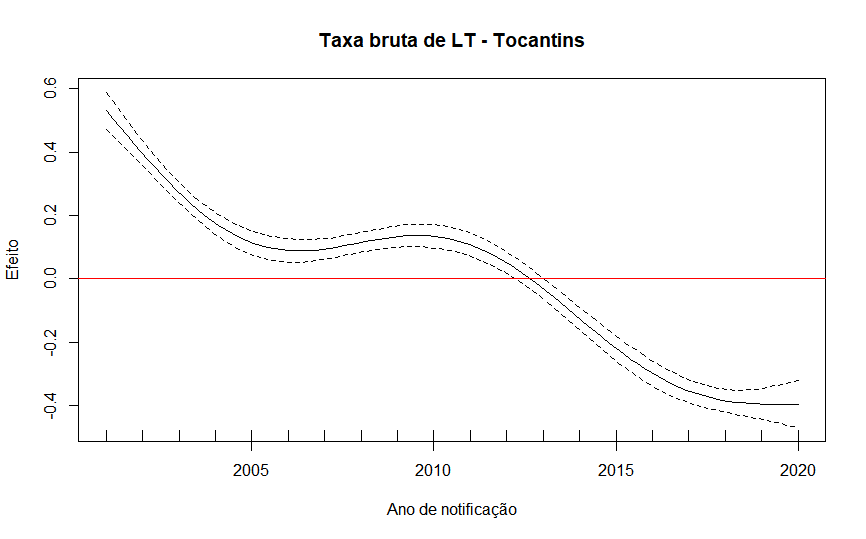
**
