# Supplementary material for: Temporal patterns, spatial risks, and characteristics of tegumentary leishmaniasis in Brazil in the first twenty years of the 21st Century
Source: PLoS Negl Trop Dis. 2023 Jun 7;17(6):e0011405. doi: 10.1371/journal.pntd.0011405 (PMC10281579; doi:10.1371/journal.pntd.0011405)

**S2 Fig. Choropleth maps of the annual incidence rates (x 100,000 inhabitants) of tegumentary leishmaniasis in the 27 federative units of Brazil between 2001 and 2020.** The maps were built using the free and open source QGIS software (https://www.qgis.org/en/site/) based on shapefiles obtained from Instituto Brasileiro de Geografia e Estatística -IBGE- (https://www.ibge.gov.br/geociencias/organizacao-do-territorio/malhas-territoriais/15774-malhas.html)


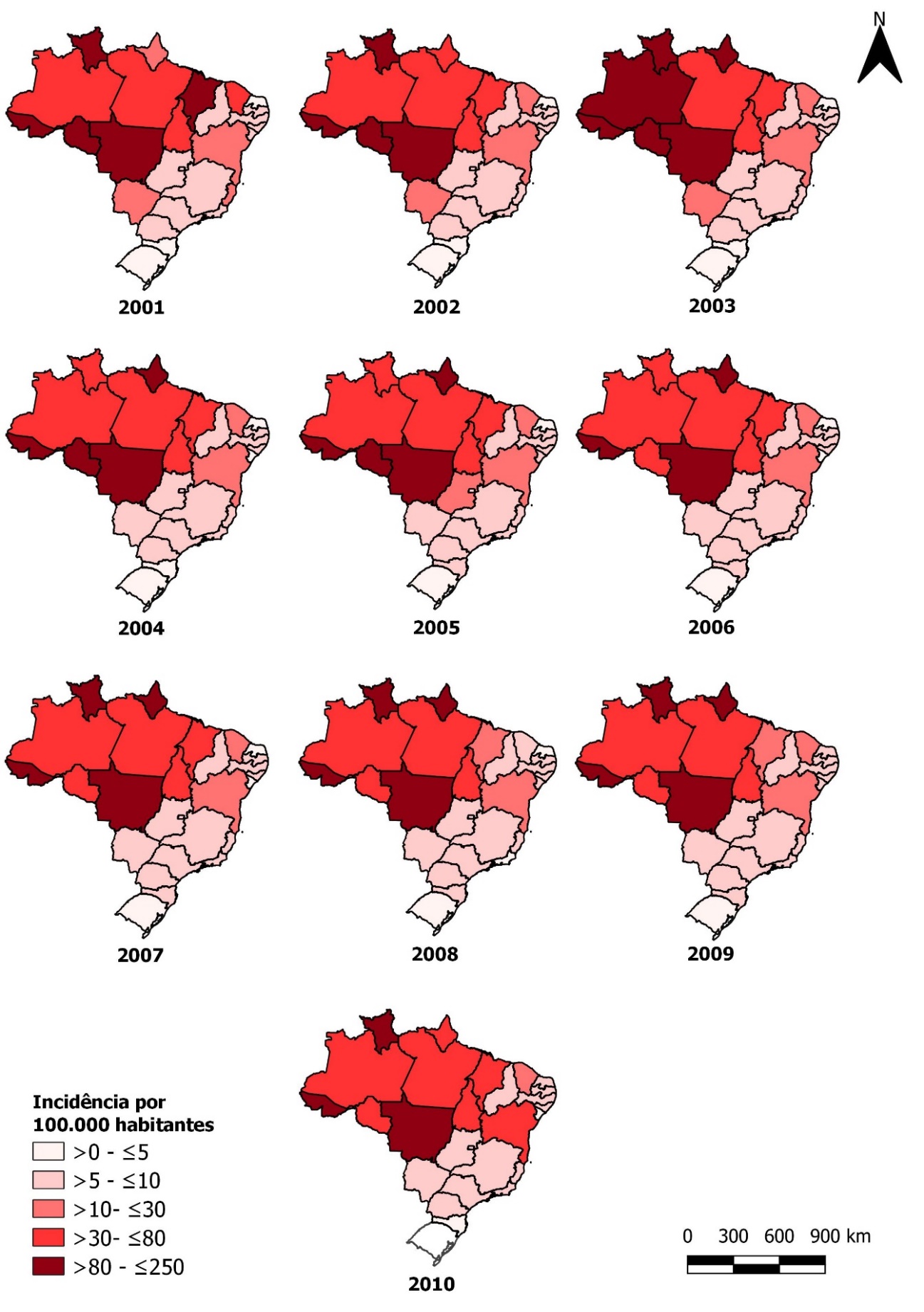


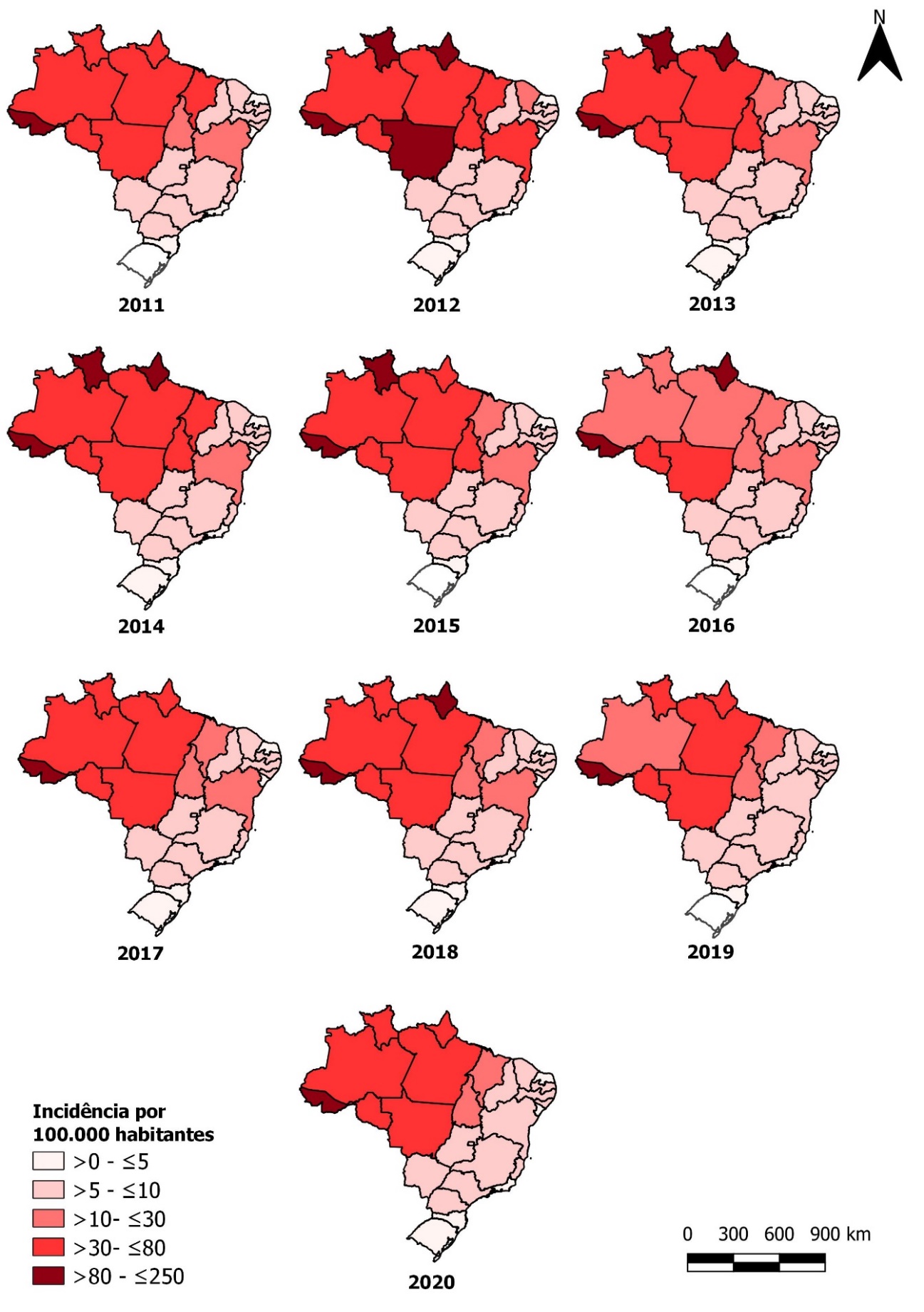

Supplement: S2 Fig — The maps were built using the free and open source QGIS software (https://www.qgis.org/en/site/) based on shapefiles obtained from Instituto Brasileiro de Geografia e Estatística -IBGE- (https://www.ibge.gov.br/geociencias/organizacao-do-territorio/malhas-territoriais/15774-malhas.html). (DOCX) [file pntd.0011405.s004.docx]
