# Supplementary material for: Temporal patterns, spatial risks, and characteristics of tegumentary leishmaniasis in Brazil in the first twenty years of the 21st Century
Source: PLoS Negl Trop Dis. 2023 Jun 7;17(6):e0011405. doi: 10.1371/journal.pntd.0011405 (PMC10281579; doi:10.1371/journal.pntd.0011405)
Supplement: S3 Fig — The maps were built using the free and open source R software (https://www.R-project.org/) based on shapefiles obtained from Instituto Brasileiro de Geografia e Estatística -IBGE- (https://portaldemapas.ibge.gov.br/portal.php#homepage). Black dots represent the centroids of Brazilian municipalities. (DOCX) [file pntd.0011405.s005.docx]

**S3 Fig. Smoothed spatial generalized additive model maps showing areas of greater risk of occurrence of tegumentary leishmaniasis in Brazil between 2001 and 2020 broken down by year.** The maps were built using the free and open source R software (https://www.R-project.org/) based on shapefiles obtained from Instituto Brasileiro de Geografia e Estatística -IBGE- (https://portaldemapas.ibge.gov.br/portal.php#homepage). Black dots represent the centroids of Brazilian municipalities.

| 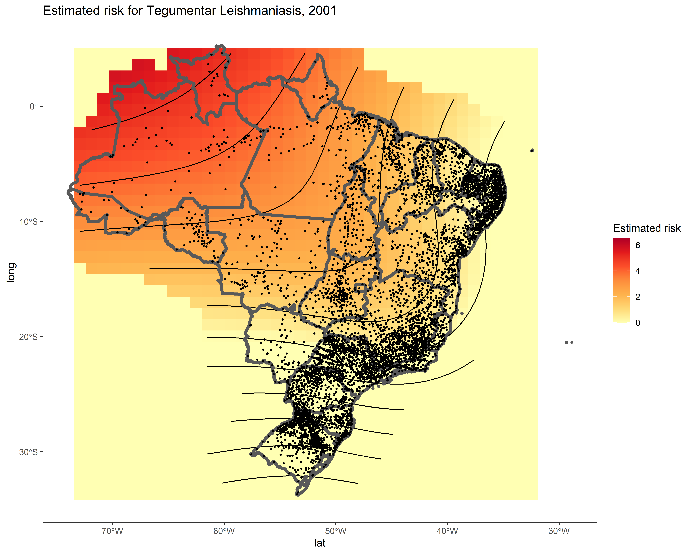 | 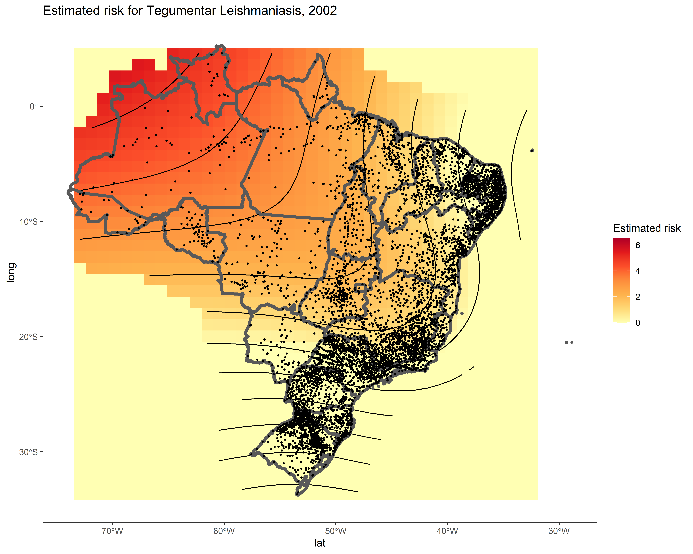 |
| --- | --- |
| 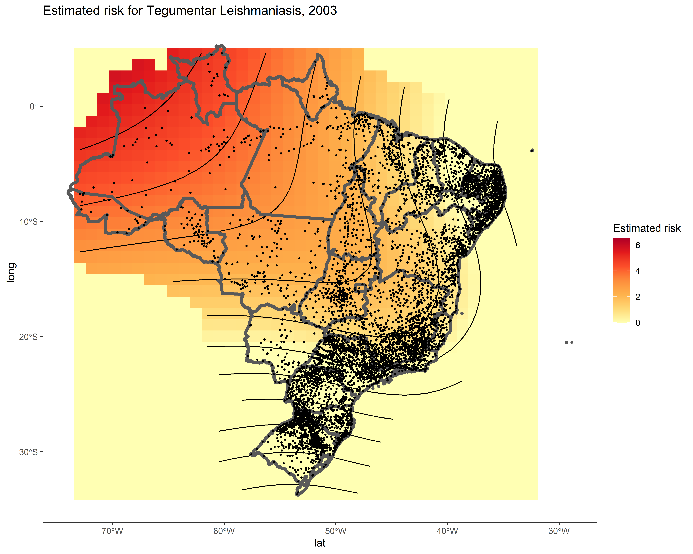  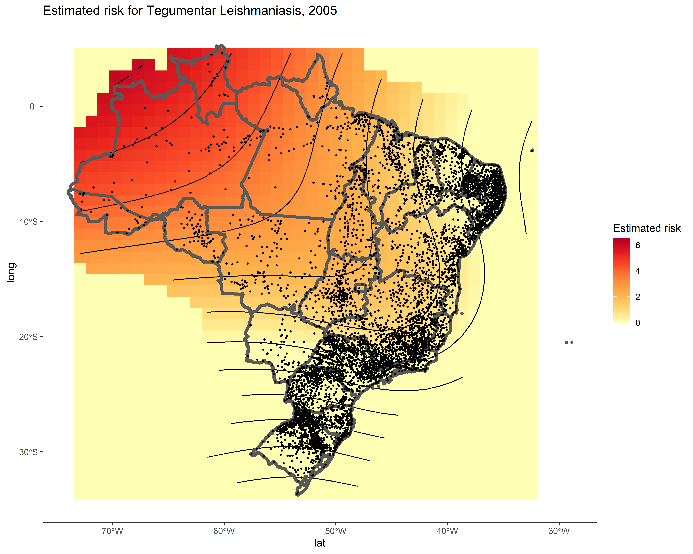 | 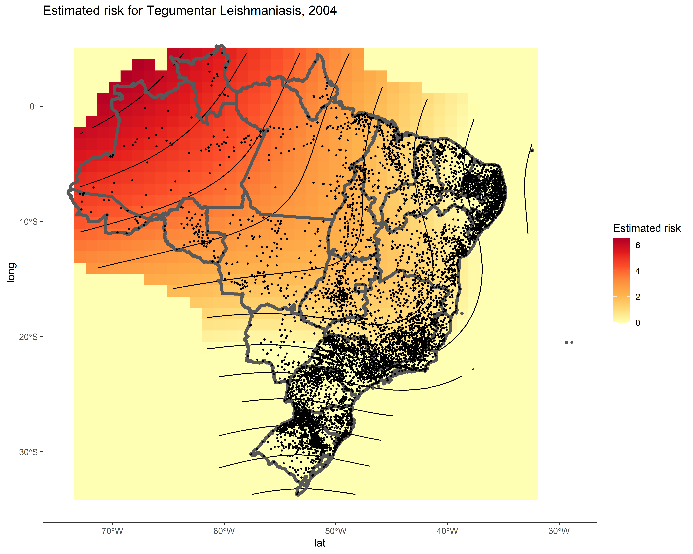  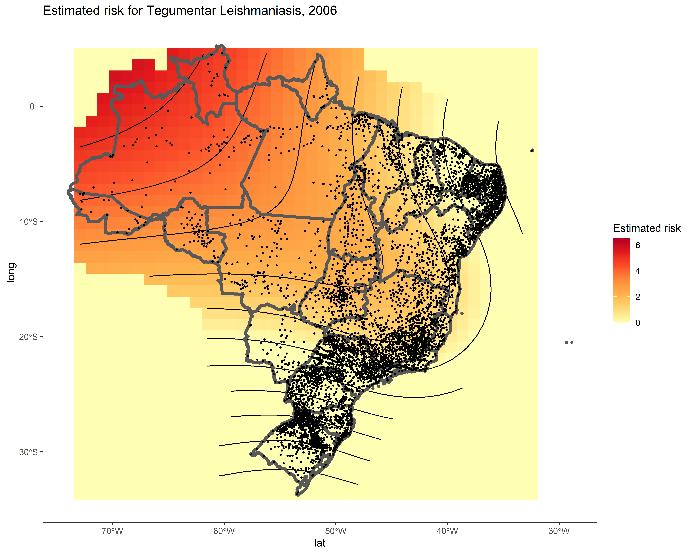 |

| 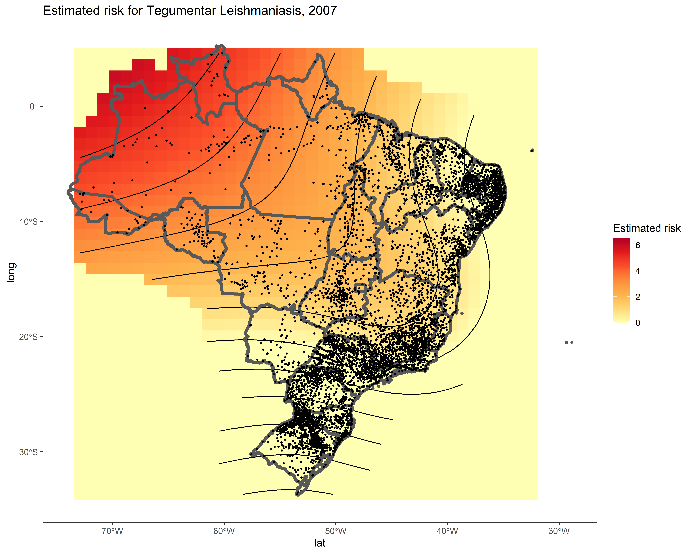 | 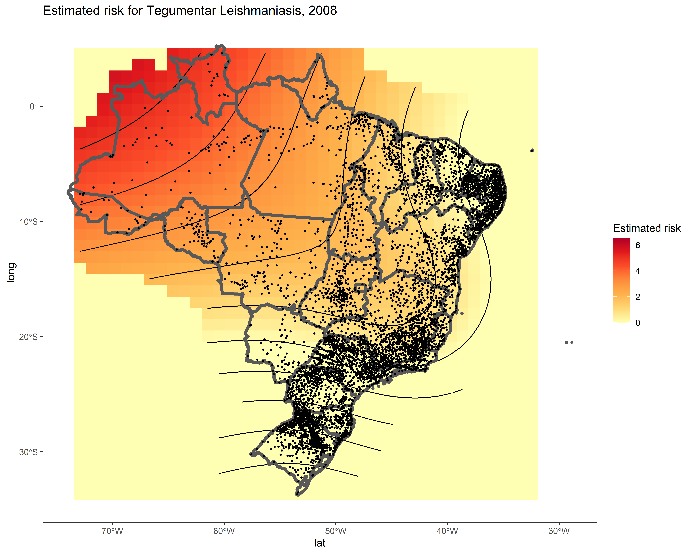 |
| --- | --- |
| 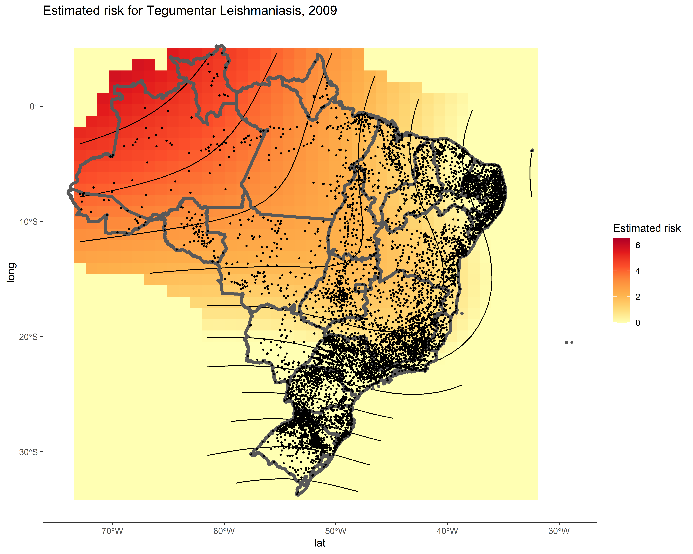  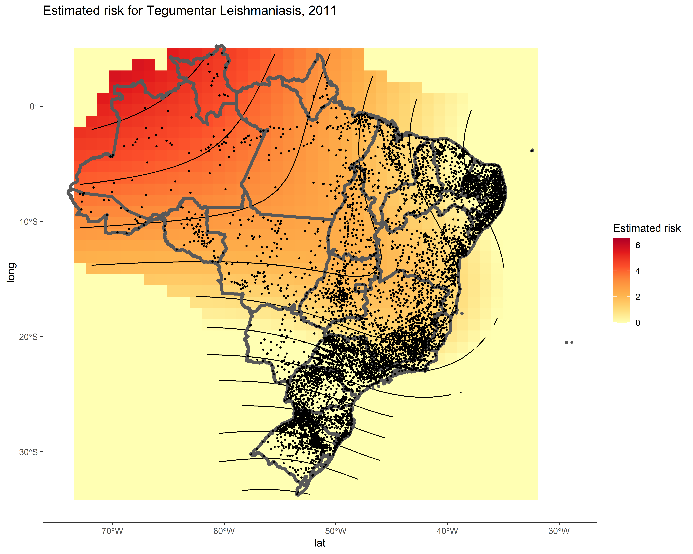 | 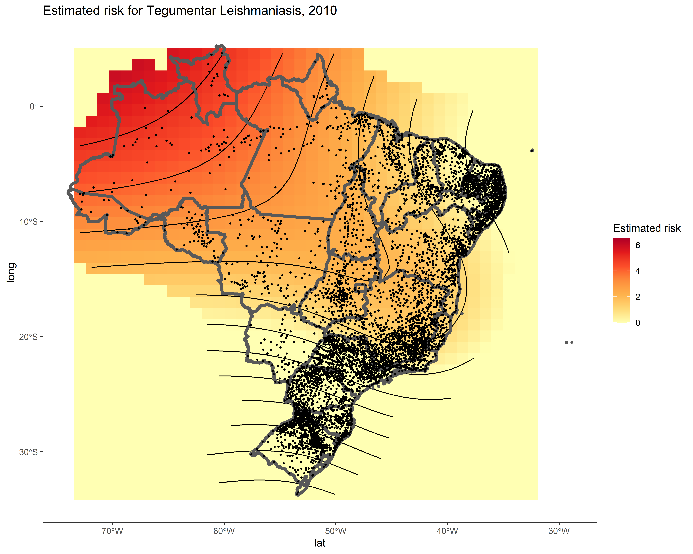  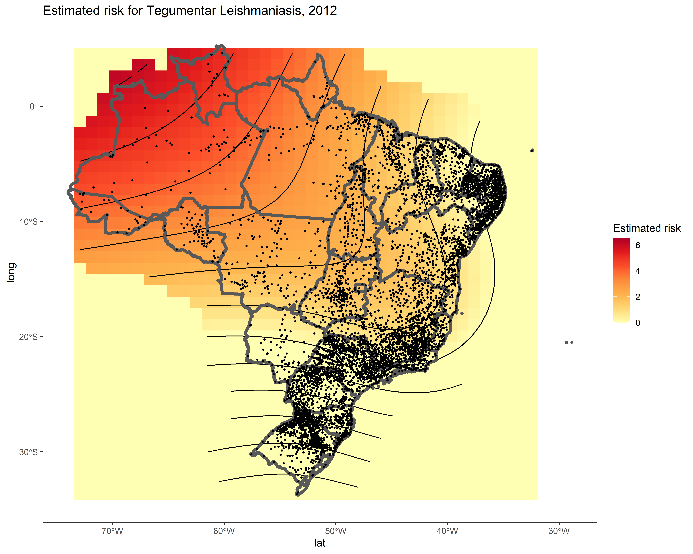 |

| 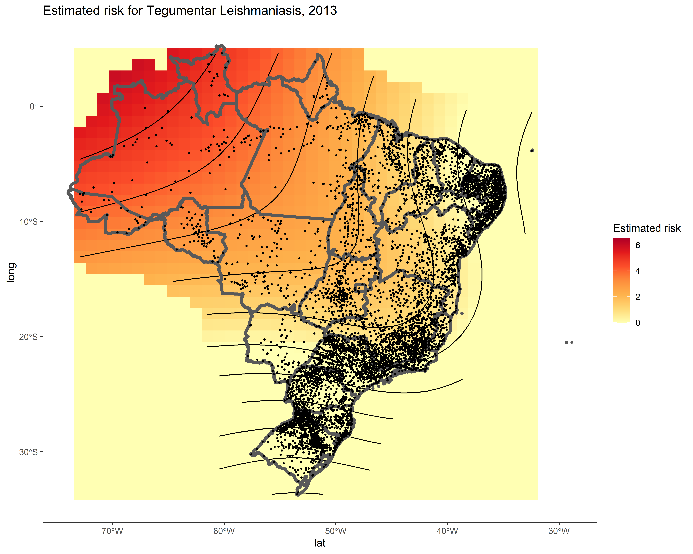 | 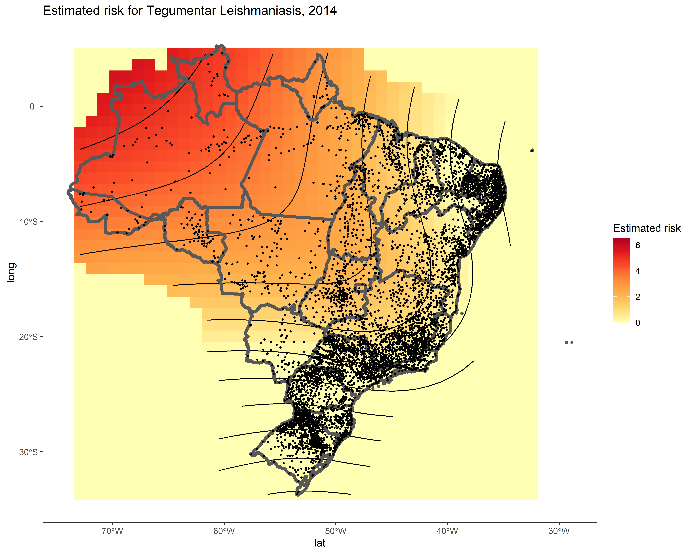 |
| --- | --- |
| 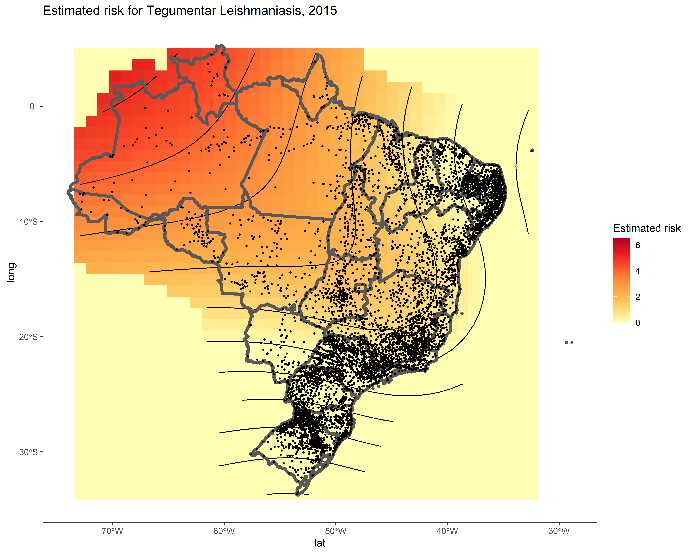  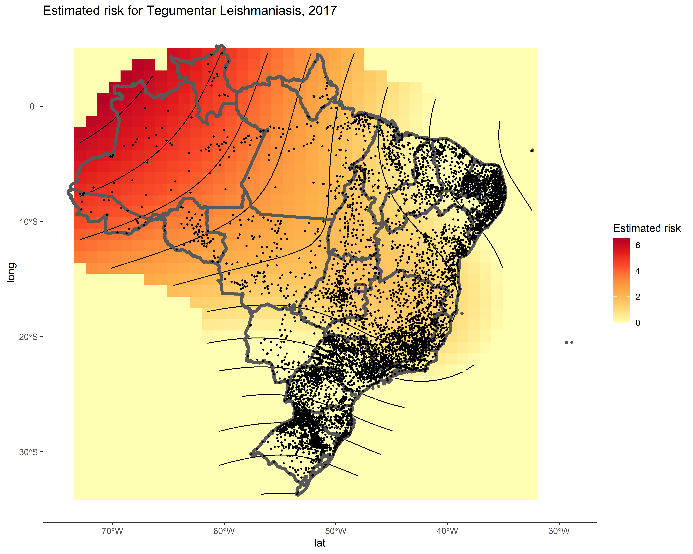 | 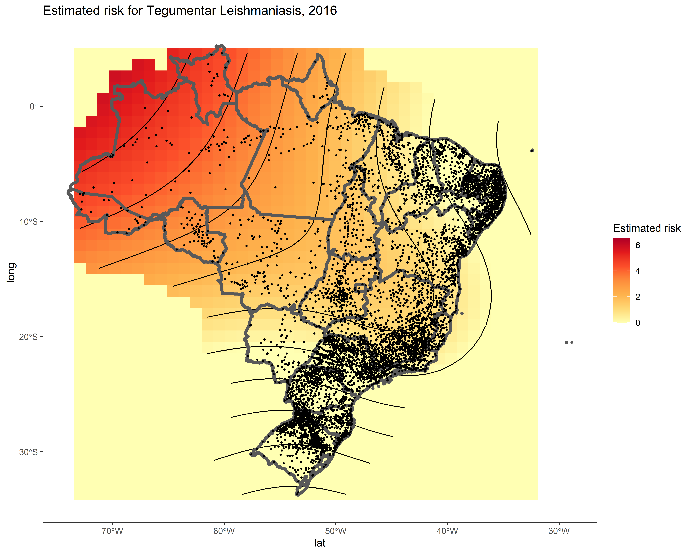  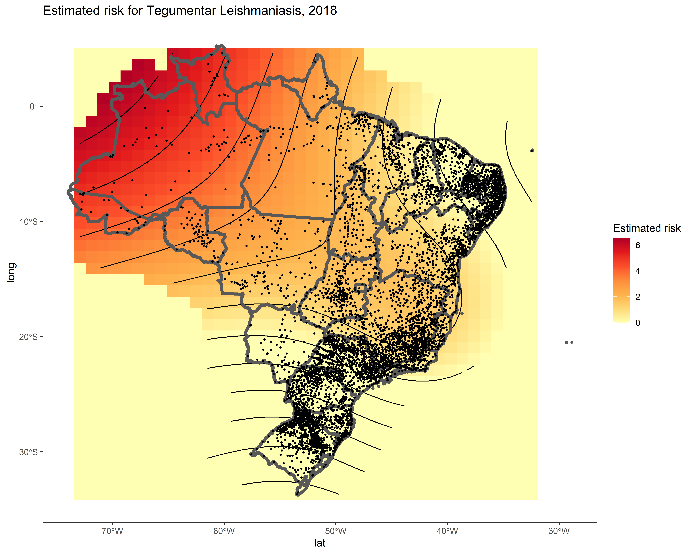 |

| 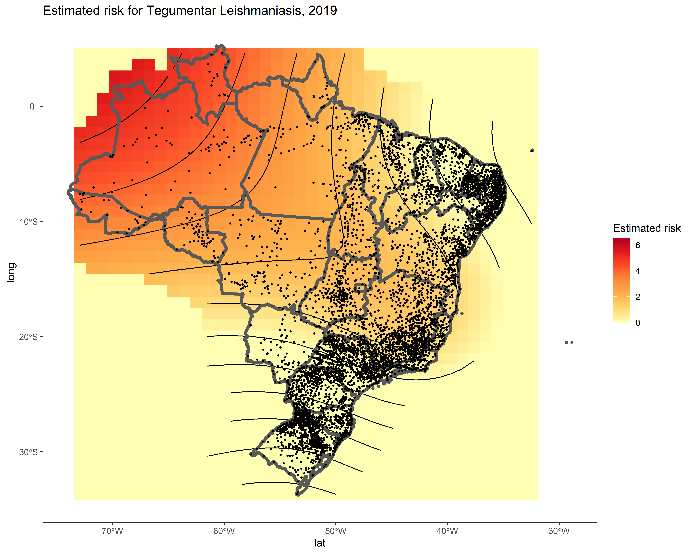 | 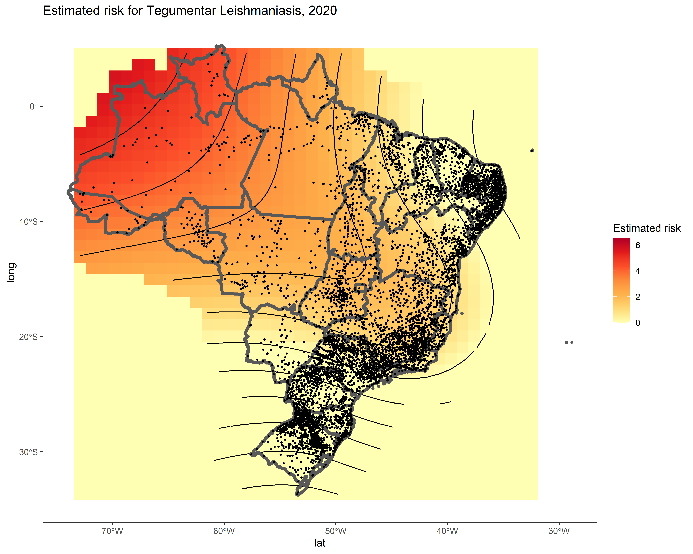 |
| --- | --- |
