# Supplementary material for: Temporal patterns, spatial risks, and characteristics of tegumentary leishmaniasis in Brazil in the first twenty years of the 21st Century
Source: PLoS Negl Trop Dis. 2023 Jun 7;17(6):e0011405. doi: 10.1371/journal.pntd.0011405 (PMC10281579; doi:10.1371/journal.pntd.0011405)
Supplement: Attachment — Submitted filename: Response letter.docx [file pntd.0011405.s007.docx]

**Response letter – PloS Neglected**

Dear Dr. Belo,

Thank you very much for submitting your manuscript "Temporal patterns, spatial risks, and characteristics of tegumentary leishmaniasis in Brazil in the first twenty years of the 21st Century" for consideration at PLOS Neglected Tropical Diseases. As with all papers reviewed by the journal, your manuscript was reviewed by members of the editorial board and by several independent reviewers. The reviewers appreciated the attention to an important topic. Based on the reviews, we are likely to accept this manuscript for publication, providing that you modify the manuscript according to the review recommendations.

We would like to thank the editor and the reviewers for the valuable questions and comments that helped us to improve our paper. We are grateful for the time and effort dedicated to reviewing our work.

We have responded to each of the comments below and made the changes indicated in the file attached.

Line numbers refer to the clean version.

Reviewer #1: Page 7, lines 115 to 116: state the year of the population estimate.

Inserted (lines 119-120).

Page 8, line 122: in the ethical considerations (lines 112 to 113) the authors inform that the information is free (that is, if open access), however, this link (http://sinan.saude.gov.br/sinan/login/login.jsf) requires login and password.

The link has been corrected (line 126).

Page 8, lines 128 to 134: for the calculation of the rates, the authors use the average population for the period, however it was not clear in this one whether the authors used the average of cases in the 20 years, or if they used the sum of cases for the period. As a suggestion, I would use the average of cases or deaths to calculate the rates, since the sum may present relatively high rates for states or administrative regions.

Considering this comment, the calculation of the average annual incidence rate was added to the text (lines 138-129 and 198) and table S1.

Reviewer #2: The study is relevant and the objective is clearly stated. However, the study design was not declared, although is clear that it consists of an ecological study.

The study design has been added to the abstract and methods (line 116).

The calculation of incidence rates must be clarified.

Considering this comment and those of reviewer 1, we have improved the explanation of the calculation of incidence rates (abstract, lines 136-139 and table S1).

Additionaly, the ranges of incidences rates should follow some pattern form OPAS, WHO or Health Ministry of Brazil.

Thank you for the suggestion. Considering that there are no predefined cutoff points for categorizing incidence rates and that PAHO and the Ministry of Health adopt a composite index that is utilized at the municipal level in Brazil, we would like to keep the ranges of incidences.

Maps were built considering incidence rates at the federative unit’s level and used the Jenks natural breaks classification method. We have added this information to the text (lines 143-145).

Reviewer #3: The objective of the study is clear and the study design is appropriate to address the purpose of the study

The study analyzed the spatial and temporal patterns of the incidences and risks of occurrence of TL across the five geopolitical regions and 27 federative units in Brazil.

Cases of TL notified between 2001 and 2020 were obtained from the Information System for Notifiable Diseases of the Brazilian Ministry of Health. Ethical approval for the project was not required because the research involved data derived from secondary sources that are freely available to the public.

Reviewer #4: This is a very interesting paper, relevant and important, for public health and neglected diseases.

We would like to thank the reviewers for their positive comments.

The results seccion need to clarify why the autors choose to estimates global inciddence, because the recidive is possible in leishmaniosis. The indicator needs the time reference.

The main objective of the study was to identify the evolution of transmission patterns of tegumentary leishmaniasis. By focusing on new cases, we believe that we were able to capture the occurrence of new transmissions more accurately and generate results that are more suitable for understanding the historical context of the disease and directing control interventions. With the reviewer's comment in mind, we have explained this rationale in the text (lines 104-107) and included the time reference for the global incidence (abstract and lines 136-139).

The registration of deaths by SINAN is subject to failure, especially since there is no death surveillance. It is more recommended to use data from the Mortality Information System (SIM) for this measure.

Thanks for the comment. Based on it, we updated the information on death numbers and mortality rates using the Mortality Information System (SIM) (lines 126-129 and S1 table).

Reviewer #1: Page 34, fig. 1: include in the map the countries that are neighbours of Brazil.

The neighboring countries have been added to Figure 1.

Pages 35, 36, 37, 38 and 39, fig. 2 and fig. 3: standardize the years of the x axis (Example: in fig. 2 is in the sequence: 2001 to 2020, while in fig.3 we have every 5 years);

The x axis of figure 2 and figure 3 was standardized.

Pages 37 and 39, fig. 4 and fig. 6: the data of the figures are already represented in table 1. Evaluate a real need for the figures.

If possible, we would like to keep Figures 4 and 6. We believe they provide visual information that enhances the understanding of the time series trajectory and complements the numerical data presented in the table.

Reviewer #2: The results must be improved. When the results are presented, the absolute and relative frequencies should be highlighted in parentesis.

Absolute and relative frequencies were added to the results section (lines 200-248).

The images in supporting information need be improved either. The incidence rates in coropleth maps should follow the ranges standardized by Health Ministry of Brazil (if exists).

As previously mentioned, there are no pre-defined cut-off points for categorizing incidence rates. PAHO and the Ministry of Health use a composite index that is applied at the municipal level in Brazil. Therefore, we would like to maintain the ranges of incidences on the choropleth maps of Brazilian states.

There are a plent of maps depicting the incidence at state level year by year. I suggest to represent moving avarages in order to reduce the amount of images. furthermore, the legend of map from description of study area is totally wrong.

Annual maps were constructed to provide a better assessment of the temporal evolution of incidence rates in Brazilian states, complementing the information that can be seen in the Joinpoint graphs (which are on an annual basis).

The legend of the study area map has been reformulated based on the corrections indicated.

Reviewer #3: The analysis used were adequate to support the conclusions of the study. The study described the clinical and epidemiological characteristics of TL cases reported in the country. Joinpoint and generalized additive models were used to establish trends in the evolution of TL during the target period. Clinical and epidemiological characteristics of TL cases were described and analyzed.

We would like to thank the reviewer for his/her positive comments.

Reviewer #1: Page 20, lines 372 to 373: in addition to database limitations, the use of data aggregated by states/municipalities may also be a limitation, given the size of municipalities in the North of Brazil, when compared to municipalities in the South/Southeast (for example). The municipalities in the North, besides being municipalities with large territorial areas, have low population indices.

We agree with the reviewer's comment. Based on it, we added this limitation to the article discussion (lines 400-402).

Reviewer #2: I suggest that authors highlight the implications of findings for epidemiologic surveillance. It was mentioned that the results could contribute to improve therapeutic actions. However, I would rather to focus on the intersectoral strategies to reduce the iniquities among the regions and the importante of adoption of spatial and temporal tools in surveillance.

Thanks for the comment. With that in mind, we have improved the conclusion of the abstract and the discussion regarding the importance of intersectoral strategies, as well as spatial and temporal analysis, in surveillance routines (lines 359-364). We also removed the mention to therapeutic actions.

Reviewer #3: The conclusions are supported by the presented data and analysis performed. Study limitations are clearly described. The main limitation refers to the quality of the information recorded in the secondary data analyzed in the study. According to the authors the findings of the study will serve to expand the understanding of the epidemiology of TL in Brazil and are highly relevant to the improvement of public health policies. Despite the general decline of TL in Brazil, several parts of the country still presented considerable numbers of cases and/or increased numbers of notifications.

We would like to thank the reviewer for his/her positive comments.

Reviewer #3: Line 77: …”significant public health problem and requires compulsory notification”

Please, add a reference on compulsory notification diseases in Brazil. The reference 6 (World Health Organization. Leishmaniasis) is about general LT information.

The reference was added (line 80).

Lines 101-103: The reference 19; Bruhn et al., is about visceral leishmaniasis. I am not sure if this reference is in accordance with the cited text.

The reference was replaced with a new one that is more closely related to the cited passage.

Please, change the name of Central-west to Midwest region in Figure 2. All the others figures and tables the name used of the region is Midwest.

Figure 2 has been corrected according to the recommendation.

Reviewer #1: Page 3, line 36: I suggest that the authors briefly inform in the method how the global incidence rate was calculated - did they use the average of cases in the period or the sum of cases?

As pointed before, we have improved the explanation of the calculation of incidence rates and added the average annual incidence rates (abstract, lines 136-139 and table S1).

Reviewer #2: The findings of sutdy are important to provide a overall scenario of TL in the last two decades. The authors could have performed cluster analysis to demonstrate the spatial correlation, but I think that the analysis carried out are sufficent to achieve the main objetive.

Reviewer #3: The study analyzed 431,885 cases of TL reported in Brazil between 2001 and 2020. The manuscript is well-written and presents interesting results on clinical and epidemiological characteristics of TL cases and spatial and temporal patterns of TL. The discussion placed this study in context and provides a thorough review of the results found in the spatial and temporal analysis and clinical and epidemiological characteristics of TL cases. Considering the relative scarcity of good quality studies on TL, I believe this article will provide an important contribution to the literature.

Once again, we would like to thank the reviewers for their positive comments.
